# Integration of early disease-resistance phenotyping, histological characterization, and transcriptome sequencing reveals insights into downy mildew resistance in impatiens

**DOI:** 10.1038/s41438-021-00543-w

**Published:** 2021-05-01

**Authors:** Ze Peng, Yanhong He, Saroj Parajuli, Qian You, Weining Wang, Krishna Bhattarai, Aaron J. Palmateer, Zhanao Deng

**Affiliations:** 1 University of Florida, IFAS, Department of Environmental Horticulture, Gulf Coast Research and Education Center, 14625 County Road 672, Wimauma, FL 33598 USA; 2 State Key Laboratory for Conservation and Utilization of Subtropical Agro-Bioresources, South China Agricultural University, 510642 Guangzhou, China; 3 Visiting scholar at University of Florida, IFAS, Department of Environmental Horticulture, Gulf Coast Research and Education Center, 14625 County Road 672, Wimauma, FL 33598 USA; 4 Key Laboratory of Horticultural Plant Biology, Ministry of Education, College of Horticulture and Forestry Sciences, Huazhong Agricultural University, 430070 Wuhan, Hubei China; 5 University of Florida, IFAS, Department of Plant Pathology, Tropical Research and Education Center, 18905 S.W. 280th Street, Homestead, FL 33031 USA; 6 Present Address: Bayer Environmental Science US, 5000 Centregreen Way, Cary, NC 27513 USA

**Keywords:** Plant breeding, Plant immunity

## Abstract

Downy mildew (DM), caused by obligate parasitic oomycetes, is a destructive disease for a wide range of crops worldwide. Recent outbreaks of impatiens downy mildew (IDM) in many countries have caused huge economic losses. A system to reveal plant–pathogen interactions in the early stage of infection and quickly assess resistance/susceptibility of plants to DM is desired. In this study, we established an early and rapid system to achieve these goals using impatiens as a model. Thirty-two cultivars of *Impatiens walleriana* and *I. hawkeri* were evaluated for their responses to IDM at cotyledon, first/second pair of true leaf, and mature plant stages. All *I. walleriana* cultivars were highly susceptible to IDM. While all *I. hawkeri* cultivars were resistant to IDM starting at the first true leaf stage, many (14/16) were susceptible to IDM at the cotyledon stage. Two cultivars showed resistance even at the cotyledon stage. Histological characterization showed that the resistance mechanism of the *I. hawkeri* cultivars resembles that in grapevine and type II resistance in sunflower. By integrating full-length transcriptome sequencing (Iso-Seq) and RNA-Seq, we constructed the first reference transcriptome for *Impatiens* comprised of 48,758 sequences with an N50 length of 2060 bp. Comparative transcriptome and qRT-PCR analyses revealed strong candidate genes for IDM resistance, including three resistance genes orthologous to the sunflower gene *RGC203*, a potential candidate associated with DM resistance. Our approach of integrating early disease-resistance phenotyping, histological characterization, and transcriptome analysis lay a solid foundation to improve DM resistance in impatiens and may provide a model for other crops.

## Introduction

Downy mildew (DM) is a destructive disease caused by obligate parasitic oomycetes from the *Peronosporaceae* family^1^. It has been a serious challenge for a wide range of cultivated crops including row crops, vegetables, fruits, and ornamental plants. DM is globally distributed and has high adaptability to new and changing environmental conditions^2^. Most DM pathogens can infect their host plant at the seedling stage, causing systemic shoot infection, whereas infection at a more mature stage may develop into localized infection patches^3^. DM can affect the leaves, flowers, fruits, and shoots of hosts and cause great economic losses. It may lead to yield losses of up to 40–80% for different crops^4,5^. Many fungicides have been developed to manage DM pathogens; however, due to genetic recombination, frequent mutations, and asexual reproduction, new DM pathogen races with higher virulence levels emerge constantly, resulting in fungicide resistance in DM pathogens and thus severely hindering the effectiveness of fungicides whose development could take many years and cost hundreds of millions of dollars^6,7^.

DM pathogens are composed of at least 300 species belonging to different genera, such as *Peronospora*, *Pseudoperonospora*, and *Plasmopara*, among which *Peronospora* is the largest genus containing more than 260 species^8^. The common DM species infecting horticultural crops include *Peronospora destructor* (onion), *Peronospora belbahrii* (basil), *Plasmopara viticola* (grape), *Pseudoperonospora cubensis* (cucurbits), *Plasmopara halstedii* (sunflower), *Peronospora effusa* (spinach), and *Bremia lactucae* (lettuce). To combat this disease, host resistance to DM has been identified in several crops and a few resistance genes have been cloned. For example, the sunflower genome contains more than 30 DM resistance genes distributed in the domesticated and wild species^9^. In lettuce, over 50 DM resistance genes have been identified and genetically characterized, among which at least 28 genes can provide high levels of resistance against DM^10^. In grapevine, 27 quantitative trait loci (QTLs) for DM resistance have been identified from various *Vitis* species, of which the locus *Rpv3* is a major determinant for DM resistance^11^. DM pathogens secrete apoplastic and cytoplasmic effector molecules upon infection that can be recognized by the proteins encoded by plant disease-resistance genes (*R*-genes), which are primarily comprised of nucleotide-binding site leucine-rich repeat (NBS-LRR) genes. Many NBS-LRR clusters have been identified in sunflower and lettuce genomic regions involved in DM resistance^12,13^. In Arabidopsis, some Toll/interleukin-1 receptor NBS-LRR (TIR-NBS-LRR) genes such as *RPP1* confer organ-specific resistance to downy mildew^14^. In spinach, NBS genes present at the *RPF1* locus contribute to resistance to *P. effusa*^15^.

Impatiens are one of the top-selling annual bedding flowers in the United States. The genus *Impatiens* (family Balsaminaceae) contains >1000 species that are widely distributed in different geographic and climatic regions, including tropical Africa, Southeast Asia, parts of Europe, and North America^16^. Among these species, *Impatiens walleriana* and *Impatiens hawkeri* are the most commonly cultivated in the world. The popularity of impatiens in the floriculture industry is attributed to the flower color diversity, profuse flowering nature, and ease of growing^17,18^. In 2018 alone, impatiens contributed a wholesale value of more than $109 million^19^. Impatiens downy mildew (IDM) caused by *Plasmopara obducens* is currently a huge threat to the impatiens industry^20^. Severe outbreaks of IDM were reported in Europe^21^, Australia^22^, and North America^23,24^, causing significant economic losses. The outbreak of IDM in the USA has caused a significant decrease of the wholesale values of impatiens from ~$150 million in 2005 down to ~$65 million in 2015^25^. IDM caused by *P. obducens* has become a major disease of *I. walleriana*. The infected plants exhibit downward leaf curling, chlorotic and downy leaves, and leaves and flowers drop, all of which may result in complete losses of the aesthetic value of impatiens cultivars^24^. Several studies reported the morphology, transmission and hosts of *P. obducens*. This pathogen develops hyaline and monopodial sporangiophores with apical branches that can produce ovoid and hyaline sporangia^26^. The pathogen is readily transmitted by wind-blown or water-splashed sporangia from which the zoospores can be released and infect impatiens under suitable temperature and relative humidity^27^. Usually, 5–14 days after pathogen infection, visible white downy symptoms could be observed on the lower leaf surfaces^27,28^. The oospores could not be observed in fresh leaves and may survive overwinter in plant debris^26,27,29^. *Plasmopara obducens* can infect a number of cultivated and wild *Impatiens* spp., including *I. walleriana*, *I. balsamina*, *I. pallida*, *I. carpensis*, and *I. glandulifera*^28,30–33^. However, *I. hawkeri* appears to be highly resistant to this disease^22^.

Management of IDM can be achieved by using preventive fungicides. Several fungicides have been used to manage this disease in impatiens production facilities. Frequent applications of these fungicides have significantly increased the production costs and caused serious concerns over pesticide pollution of the environment. Moreover, few fungicides are available for use to manage this disease in the landscape (public or residential) and indoor exhibitions where impatiens are grown^34,35^. Developing and using disease-resistant cultivars have proven to be an effective, economic, and sustainable approach to managing devastating diseases in crops if genetic disease resistance can be found or developed. For example, disease-resistant cultivars have played an essential and critical role in controlling grapevine DM caused by *P. viticola*, which is in the same genus with the IDM pathogen *P. obducens*^36^. To develop disease-resistant cultivars, disease screening is essential and most critical. First, germplasm accessions, as many as possible, need to be screened to discover useful sources of disease resistance. Then, large breeding populations, generation after generation, need to be screened to identify the resistant progeny. Thus, effective and efficient disease screening or resistance phenotyping techniques frequently determine the success of plant disease-resistance breeding in many crops. IDM resistance has become the most important breeding objective in impatiens in the world; the development of effective and efficient IDM screening techniques would be of tremendous value to this important crop.

RNA-sequencing (RNA-Seq) technology has been used to identify genes potentially involved in DM resistance in horticultural plants, including lettuce, grapevine, spinach, and impatiens^37–42^. Previously, two de novo RNA-Seq comparative analyses of impatiens have identified some differentially expressed genes, including a couple of NBS-LRR genes for IDM resistance and candidates for IDM susceptibility^41,42^. However, in the absence of a reference genome, an accurate transcriptome and full lengths of the candidate genes are hardly achievable using short reads of RNAs based on the Illumina sequencing platform. Isoform sequencing (Iso-Seq), an advanced technique based on the single-molecule real-time (SMRT) sequencing platform and long reads of RNAs, has facilitated retrieval of full-length transcripts, assembly of high-quality reference transcriptomes, and discovery of splicing events and novel transcripts^43^. With Iso-Seq, each mRNA-derived cDNA molecule in a transcriptome is sequenced multiple rounds, resulting in high-quality full-length cDNA or corresponding mRNA sequences. Currently, a reference-level high-quality transcriptome for impatiens is not available. Study of the gene expression profiles at different developmental stages for different resistant and susceptible plants can function as a model to study DM-plant interaction at the transcriptome level and to uncover the plant–pathogen dynamics during resistance development.

In this study, the disease responses of 32 impatiens cultivars, including 16 *I. walleriana* and 16 *I. hawkeri* cultivars, were investigated at the cotyledon, first/second pair of true leaf, and mature plant stages, aiming to establish a system for early and rapid screening and phenotyping of impatiens for IDM resistance. DM pathogen growth and development in cotyledons and leaves were examined histologically, revealing the IDM-resistance mechanisms in *I. hawkeri*. Moreover, full-length transcriptome sequencing combined with RNA-Seq was applied to investigate transcriptome dynamics for three representative cultivars showing different resistance and susceptibility at cotyledon and true leaf stages. The transcriptome comparisons between IDM-resistant and susceptible cultivars and tissues revealed a core set of genes, including three *R*-genes potentially involved in IDM resistance in impatiens. Results from this study have provided very useful genomic resources and laid a solid foundation for future studies to implement genomics-assisted breeding of impatiens for IDM resistance and to identify and clone the IDM-resistance genes in impatiens in the future.

## Results

### Responses of 32 cultivars to natural downy mildew pathogen infection

In total, 16 cultivars of *I. walleriana* and 16 cultivars of *I. hawkeri* (Table 1) were evaluated in the field for their response to IDM at the mature stage. On December 28, 2014 (206 days after planting (DAP)) (average temperature 20.51 °C, relative humidity 88%, rainfall 0 cm)^44^, “Balance Orange” (BO) and “Super Elfin Pink” (SEP) of *I. walleriana* first showed IDM white sporulation on the abaxial side of foliage. Within 3 days, all plants of *I. walleriana*, sooner or later, showed similar IDM symptoms (Table 1). Infected impatiens plants showed chlorotic and downward-curling leaves, followed by leaf and flower dropping, complete defoliation, and plant collapse within a 7-week period. All plants died before February 16, 2015 (256 DAP), indicating all these *I. walleriana* cultivars are highly susceptible to IDM. By contrast, all plants of *I. hawkeri* cultivars did not show any IDM disease symptoms through the field experiment, suggesting that they possess strong resistance to IDM at the mature plant stage (Table 1).

**Table 1:** Evaluation of *I. walleriana* and *I. hawkeri* cultivars to impatiens downy mildew caused by *Plasmopora obducens* under natural disease pressures in the field and artificial inoculation

Plant ID	Species	Cultivar name	Field evaluation of mature plants	After inoculation with *P. obducens* sporangia		
				Cotyledon stage	First/second pair of true leaf stage	Mature plant stage
APDO	*I. walleriana*	Accent Premium Deep Orange	S	S	S	S
APR	*I. walleriana*	Accent Premium Rose	S	S	S	S
APV	*I. walleriana*	Accent Premium Violet	S	S	S	S
APW	*I. walleriana*	Accent Premium White	S	S	S	S
BO	*I. walleriana*	Balance Orange	S	S	S	S
BP	*I. walleriana*	Balance Pink	S	S	S	S
BR	*I. walleriana*	Balance Rose	S	S	S	S
BW	*I. walleriana*	Balance White	S	S	S	S
SEP	*I. walleriana*	Super Elfin Pink	S	S	S	S
SER	*I. walleriana*	Super Elfin Red	S	S	S	S
SEV	*I. walleriana*	Super Elfin Violet	S	S	S	S
SEW	*I. walleriana*	Super Elfin White	S	S	S	S
XP	*I. walleriana*	Xtreme Punch	S	S	S	S
XR	*I. walleriana*	Xtreme Red	S	S	S	S
XV	*I. walleriana*	Xtreme Violet	S	S	S	S
XW	*I. walleriana*	Xtreme White	S	S	S	S
DB	*I. hawkeri*	Divine Burgundy	R	S	R	R
DBP	*I. hawkeri*	Divine Blue Pearl	R	S	R	R
DCR	*I. hawkeri*	Divine Cherry Red	R	S	R	R
DL	*I. hawkeri*	Divine Lavender	R	R	R	R
DO	*I. hawkeri*	Divine Orange	R	S	R	R
DOB	*I. hawkeri*	Divine Orange Bronze Leaf	R	S	R	R
DP	*I. hawkeri*	Divine Pink	R	S	R	R
DSB	*I. hawkeri*	Divine Scarlet Bronze Leaf	R	S	R	R
DSR	*I. hawkeri*	Divine Scarlet Red	R	S	R	R
DV	*I. hawkeri*	Divine Violet	R	S	R	R
DWB	*I. hawkeri*	Divine White Blush	R	S	R	R
FLR	*I. hawkeri*	Florific Lavender	R	R	R	R
FR	*I. hawkeri*	Florific Red	R	S	R	R
FSO	*I. hawkeri*	Florific Sweet Orange	R	S	R	R
FV	*I. hawkeri*	Florific Violet	R	S	R	R
FW	*I. hawkeri*	Florific White	R	S	R	R

All infected *I. walleriana* plants in the field showed chlorotic and downward-curling leaves with white downy mildew sporulation (growth) on the lower surface at the early infection stage and then followed by leaf and flower drops and plant collapsing in a seven-week period. No disease symptom was observed on *I. hawkeri* plants during field experiments. Details of disease incidence for inoculation experiments were described in Supplementary Table S1. “S” indicates susceptibility to impatiens downy mildew; “R” indicates resistance to impatiens downy mildew.

### Phenotyping for downy mildew resistance at the earliest plant growth stages

**Fig. 1:**
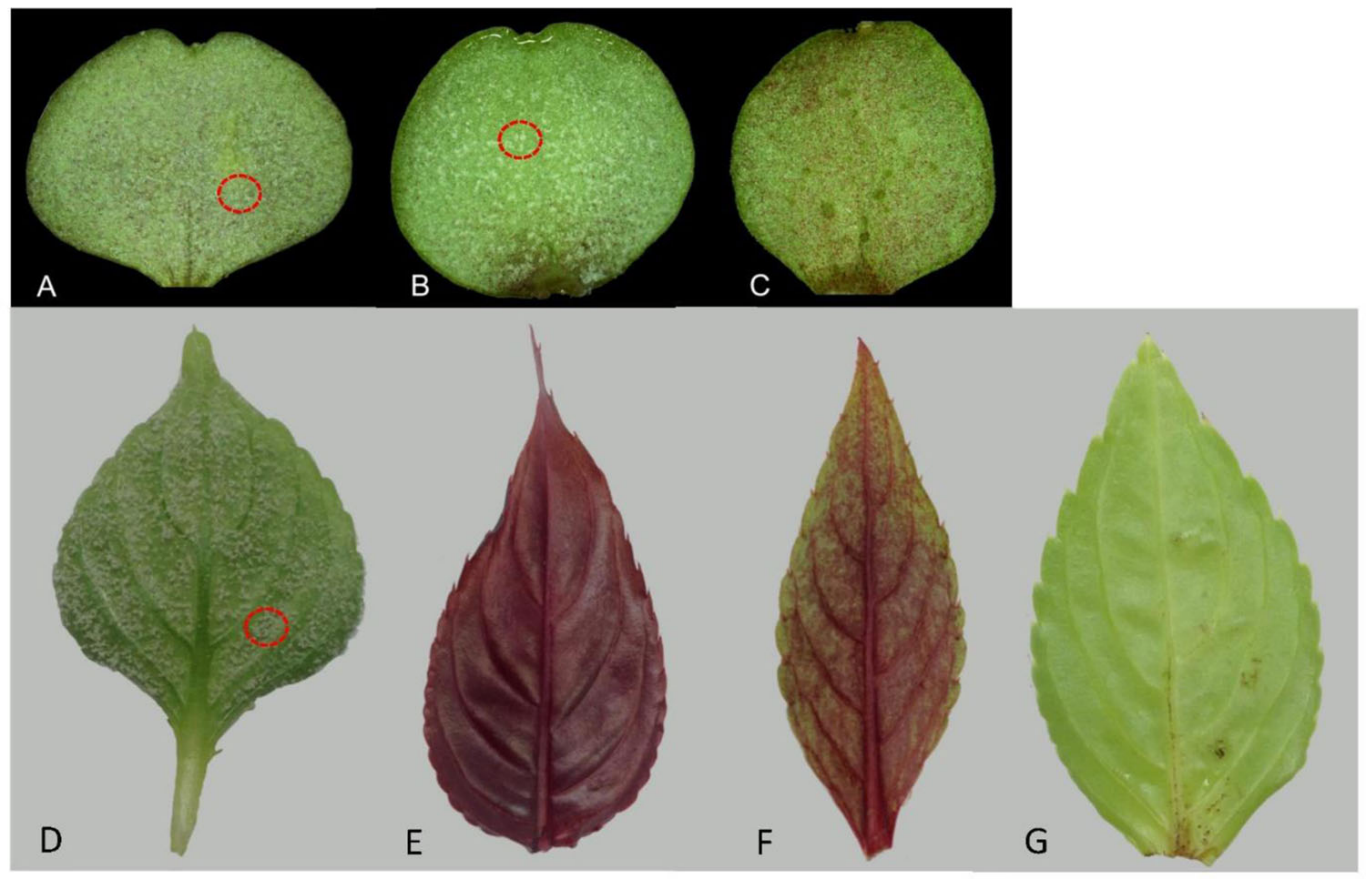
Different responses of *I. walleriana* and *I. hawkeri* to *P*. *obducens* (the causal agent of impatiens downy mildew) spores inoculated at the cotyledon or the second pair of true leaf stage. Photos were taken 10 days post inoculation. **A** Cotyledon of *I. walleriana* cv. “Super Elfin Red”; **B** cotyledon of *I. hawerki* cv. “Divine Orange Bronze Leaf”; **C** cotyledon of *I. hawerki* cv. “Florific Lavender”; **D** second true leaf of *I. walleriana* cv “Super Elfin Red”; **E** second true leaf of *I. hawerki* cv. “Divine Orange Bronze Leaf”; **F** second true leaf of *I. hawerki* cv. “Florific Lavender”; and **G** second true leaf of *I. hawerki* cv. “Divine White Blush”. The red circles highlight the white downy mildew sporulation

To develop an effective early and rapid phenotyping system, we inoculated young plants of these 32 cultivars at their earliest growth stages (cotyledon and first/second pair of true leaf stages) as well as mature leaves using two inoculation methods (*P*. *obducens* spores applied to the abaxial or adaxial side of the cotyledons, first/second pair of true leaves, and mature leaves). Results showed that all *I. walleriana* cultivars were highly susceptible to IDM at all these stages (Table 1 and Supplementary Table S1). Typical white downy mildew sporulation was evident and profuse on the abaxial side of cotyledons and true leaves for all *I. walleriana* cultivars (Fig. 1A, D). All 16 cultivars of *I. hawkeri* showed resistance to IDM at the first/second pair of true leaf stage (Fig. 1E, F), consistent with typical plant responses of these cultivars to IDM at the mature stage. These results indicate that young impatiens plants at their first true leaf stage have developed resistance to IDM and are ready for IDM disease screening or phenotyping for IDM resistance.

Interestingly, *I. hawkeri* plants at the cotyledon stage exhibited different responses to inoculated *P*. *obducens* spores (*P* < 0.05). When inoculated on the abaxial side of cotyledons, “Divine Orange Bronze Leaf” (DOB) (Fig. 1B), “Divine Burgundy” (DB), “Divine Orange” (DO), and “Florific Violet” (FV) were susceptible to IDM with a disease incidence index at 0.67, 0.61, 0.56, and 0.50 at 10 days post inoculation (dpi), respectively (Supplementary Table S1). “Florific Sweet Orange” (FSO), “Divine White Blush” (DWB), “Florific White” (FW), and “Divine Violet” (DV) were also susceptible to DM, but with a lower disease incidence index (≤0.14). Most importantly, “Florific Lavender” (FLR) (Fig. 1C) and “Divine Lavender” (DL) showed strong resistance to IDM even at this early stage. There were no significant differences between 10 and 20 dpi across all cultivars, except that “Divine Pink” (DP) and DO showed significant differences when inoculated on the abaxial and adaxial side, respectively (Supplementary Table S1). When the adaxial side was inoculated, the disease incidence index was lower than that of the abaxial side (*P* < 0.05) for most cultivars except DV, FW (higher at the adaxial side). Again, DL and FLR showed strong resistance to IDM after their cotyledons were inoculated on either side, with zero disease incidence (Supplementary Table S1). On the other hand, DOB and DB consistently showed susceptibility to IDM when either side of the cotyledon was inoculated. These results indicated that 14 *I. hawkeri* cultivars were susceptible to IDM at the cotyledon stage and turned resistant starting at the true leaf stage, while two *I. hawkeri* cultivars (DL and FLR) expressed strong resistance to IDM starting at the cotyledon stage.

**Fig. 2:**
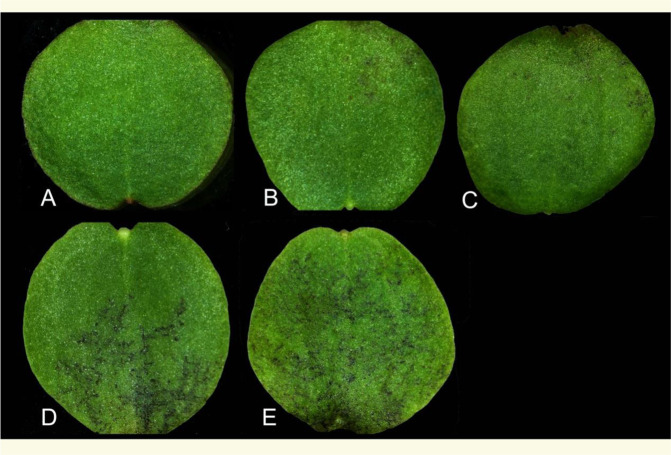
Different severity of black spots on the adaxial surface of cotyledons of *I. hawkeri* plants inoculated with *P. obducens* spores. Based on the coverage of black spots on the inoculated cotyledon, black spot severity was scored in a scale of 0 to 4. **A** scale 0, no black spots; **B** scale 1, black spots covering less than ¼ of the cotyledon; **C** scale 2, black spots covering ¼ to ½ of the cotyledon; **D** scale 3, black spots covering ½ to ¾ of the cotyledon; and **E** scale 4, black spots covering more than ¾ of the cotyledon

An interesting feature was observed on the adaxial and abaxial surfaces of inoculated cotyledons of *I. hawkeri*, but not on cotyledons of *I. walleriana* cultivars. During incubation after inoculation with *P. obducens* spores, irregular black “spots” and “specks” began to develop on cotyledon surfaces. Their occurrence varied among *I*. *hawkeri* cultivars but seemed to be from necrotic cells. For simplicity and convenience, we tentatively called them as “black spots”. To quantify the severity of black spots on cotyledons, we developed a black spot severity scale and calculated a black spot severity index (Fig. 2 and Supplementary Table S2). Black spot severity index for all *I*. *hawkeri* cultivars, except for “Divine Blue Pearl” (DBP), seemed to remain unchanged from 10 to 20 dpi. The cultivars DOB and DWB exhibited a higher black spot severity index than other cultivars at 10 dpi, with an average index of 2.18 and 2.08, respectively (Supplementary Table S2). Five cultivars, including FSO, FW, FLR, “Divine Cherry Red” (DCR), and DP showed a lower black spot severity index (<0.60). A similar trend was observed on the adaxial side of inoculated cotyledons, except that black spot severity was generally lower. The black spot severity index at 10 dpi appeared to be less than that at 20 dpi, but significant differences were not detected. At the first and second pair of true leaf stages, all *I. hawkeri* cultivars showed resistance to IDM and had no black spots, except DWB displaying small black spots on the leaf surface (Fig. 1G). When the IDM disease incidence indices (Supplementary Table S1) and the black spot severity indices of the 16 *I*. *hawkeri* cultivars (Supplementary Table S2) were examined, the Pearson Correlation Coefficient was 0.66 (abaxial, 10 dpi), 0.53 (abaxial, 20 dpi), 0.50 (adaxial, 10 dpi), and 0.51 (adaxial, 20 dpi), respectively, with an average of 0.55. Therefore, in general, the IDM disease incidence index had a moderate level of positive relationship with black spot severity. For example, the cotyledons of DOB exhibited high IDM incidence and severe black spotting after inoculation with *P. obducens*. However, there were some exceptions, for example, the cotyledons of DWB showed low IDM incidence yet severe black spotting. These results show that impatiens plants at their cotyledon stage may not express their typical mature plant resistance to *P*. *obducens*. However, if they do express resistance at this stage, they retain the resistance to IDM through their growth and developmental stages.

### Histological characterization of the disease-resistance response

Three cultivars, *I. walleriana* SER, *I. hawkeri* DOB and FLR, were selected for detailed histological characterization. In the above-described phenotyping experiments, they showed contrasting IDM-resistance responses: SER—susceptible at cotyledon, first/second pair of true leaf, and mature plant stages; DOB—susceptible at the cotyledon stage and resistant at the first/second pair of true leaf and mature plant stages; and FLR—resistant at cotyledon, first/second true leaf, and mature plant stages. Their cotyledons and true leaves were excised, inoculated with *P*. *obducens* sporangia, and cultured on 1% water agar. White downy mildew sporulation was evidently observed on the abaxial surface of cotyledons of SER and DOB at 4 dpi and became more and more massive at 6, 8, and 10 dpi. By contrast, only tiny mildew sporulation was observed on the cotyledons of FLR until 8 or 10 dpi, and the area of DM was very limited and not enlarging. When true leaves were inoculated, white mildew sporulation was only observed on the abaxial leaf surface of SER at 6 dpi but not on leaf surfaces of DOB and FLR. These results confirmed that the cotyledons of SER and DOB and true leaves of SER were susceptible to IDM, while the cotyledons of FLR and true leaves of DOB and FLR were resistant to IDM.

The sporangia density on the cotyledons and true leaves of SER, DOB, and FLR at 4, 6, 8, and 10 dpi was determined (Table 2). No *P. obducens* sporangia were observed on the leaves of DOB and FLR, and a small number of sporangia could be counted on the cotyledons of FLR at 8 and 10 dpi. On the other hand, the sporangia density on the cotyledon of SER reached 3.03 × 10^3^ sporangia cm^−2^ at 4 dpi, approximately three times higher than that of DOB, and at this time point, no white mildew growth could be observed on the leaves of SER yet. The sporangia density on cotyledons and leaves of SER at 10 dpi were 480 × 10^3^ and 404 × 10^3^ sporangia cm^−2^, respectively, and almost two times greater than that on the cotyledons of DOB. It showed that the susceptible levels of cotyledons and leaves of SER to *P. obducens* were greater than that of the cotyledons of DOB.

**Table 2:** Sporangia density (mean value ± standard deviation; 10^3^ cm^−2^) of *P. obducens* on the adaxial side of cotyledons and true leaves at different time points, days post inoculation (dpi)

Cultivar	Organ	4 dpi	6 dpi	8 dpi	10 dpi
SER	Cotyledon	3.03 ± 0.64a	390.00 ± 33.60a	470.00 ± 124.21a	480.00 ± 141.82a
DOB	Cotyledon	1.13 ± 0.17b	149.00 ± 23.40b	220.00 ± 47.81b	218.00 ± 24.84c
FLR	Cotyledon	0.00 ± 0.00c	0.00 ± 0.00d	0.34 ± 0.44c	0.63 ± 0.72d
SER	True leaf	0.00 ± 0.00c	21.00 ± 11.26c	178.00 ± 56.20b	404.00 ± 83.80b
DOB	True leaf	0.00 ± 0.00c	0.00 ± 0.00d	0.00 ± 0.00c	0.00 ± 0.00d
FLR	True leaf	0.00 ± 0.00c	0.00 ± 0.00d	0.00 ± 0.00c	0.00 ± 0.00d

*SER* *I. walleriana* Super Elfin Red, *DOB* *I. hawkeri* Divine Orange Bronze Leaf, *FLR* *I. hawkeri* Florific Lavender. Sporangia densities = total number of sporangia/area of the cotyledon or leaf segment sampled (×10^3^ cm^−2^). For each time point, eight pieces of cotyledon or leaf segments were sampled, and this was repeated three times. Different lowercase letters in the same columns indicate significant differences at *P* < 0.05 by Duncan’s new multiple range method

**Fig. 3:**
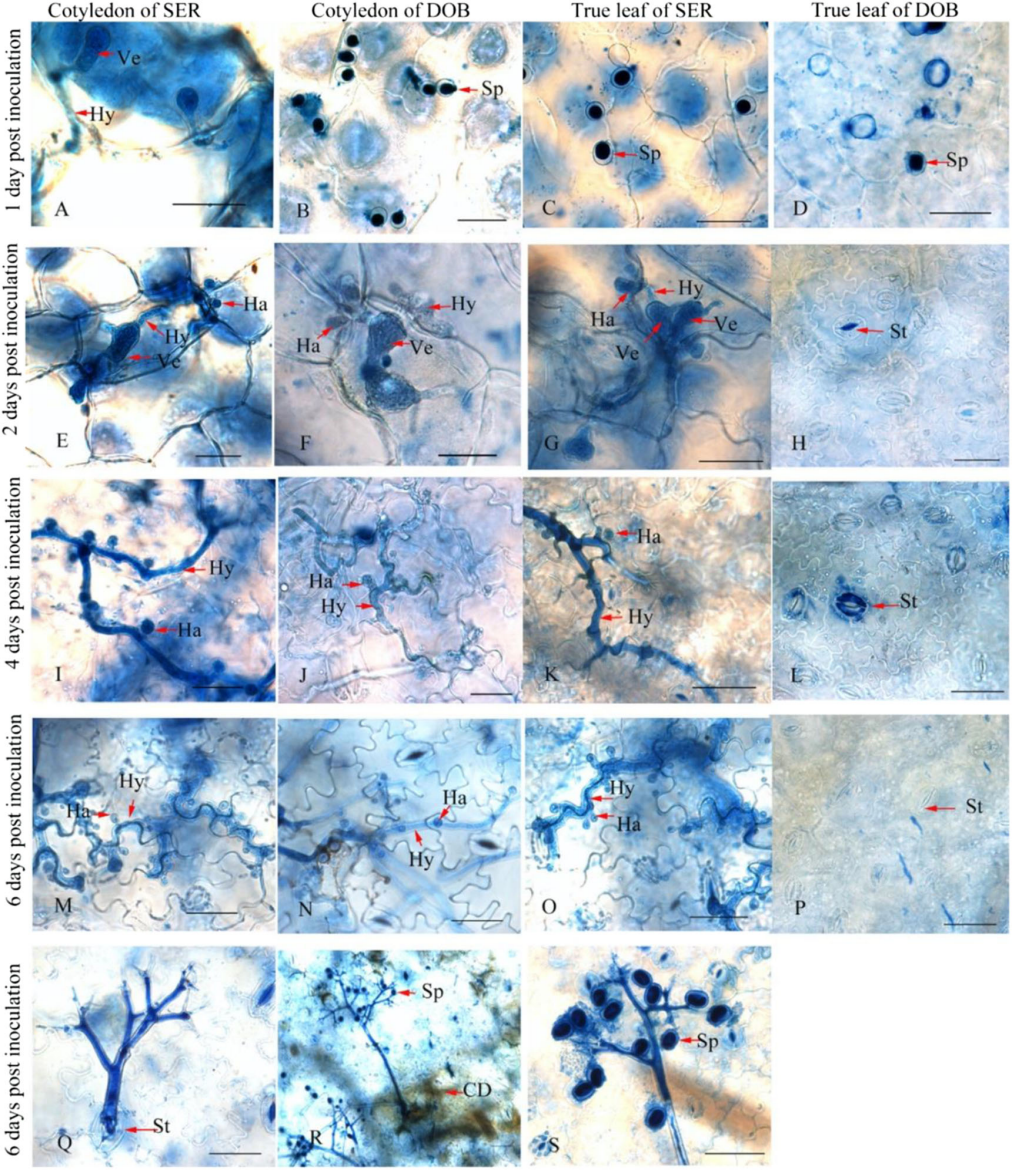
Growth and sporulation of *P. obducens* inside or on cotyledons and true leaves of *I. walleriana* cv. “Super Elfin Red” (SER) and *I. hawkeri* cv. “Divine Orange Bronze Leaf” (DOB) at 1, 2, 4, and 6 days post inoculation (dpi) as revealed by trypan blue staining. **A**–**D** vesicles and hyphae were observed inside the cotyledon of SER at 1 dpi (**A**), and sporangia attached on the surface of cotyledons of DOB (**B**) and true leaves of SER (**C**) and DOB (**D**). **E**–**G** Vesicles, hyphae, and haustoria were formed inside cotyledons of SER (**E**) and DOB (**F**) and true leaves of SER (**G**) at 2 dpi. **I**–**K** Long hyphae and haustoria were observed inside cotyledons of SER (**I**) and DOB (**J**), and true leaves of SER (**K**) at 4 dpi. **M**–**O** More hyphae and haustoria were seen inside cotyledons of SER (**M**) and DOB (**N**), and true leaves of SER (**O**) at 6 dpi. **Q**–**S** Monopodially branched sporangiophores emerged through stomata and sporangia were borne on the tips of sporangiophore branches. In **Q**, the sporangia were washed off during trypan blue staining. In **R**, the brown coloration indicated host plant cell death. **H**, **L**, **P** No development of vesicles, hyphae, or haustoria inside true leaves of DOB at 2, 4, and 6 dpi. CD cell death, Sp sporangia, Ve vesicle, Hy hypha, Ha haustorium, St stomata, Scale bars: 50 μm

To assess *P. obducens* development on impatiens, inoculated cotyledon and true leaf segments of SER, DOB, and FLR were stained with trypan blue at 1, 2, 3, 4, 5, and 6 dpi and examined microscopically. On cotyledons of SER and DOB and true leaves of SER, similar *P. obducens* development was observed. *Plasmopara obducens* sporangia first penetrated into the adaxial leaf surface (Fig. 3B, C) and then formed vesicles, intercellular hyphae, and haustoria (Fig. 3A, E, F, G) at 1 or 2 dpi. The vesicles development in cotyledons of SER was earlier than that in cotyledons of DOB and true leaves of SER. Evident hyphae and haustoria growth were seen at 4 dpi (Fig. 3I, K) and 6 dpi (Fig. 3M–O). Monopodially branched sporangiophores first emerged from stomata at 4 dpi on cotyledons of SER and DOB, and then profuse sporangiophores and sporulation were seen on cotyledons of SER and DOB and true leaf of SER at 6 dpi (Fig. 3Q–S and Table 2). On cotyledons of DOB, apparent cell death response could be observed (Fig. 3R). On the true leaves of FLR and DOB, inoculated sporangia were observed on the adaxial leaf surface (Fig. 3D), but the development of new vesicles, hyphae, or haustoria was not seen (Fig. 3H, L, P). Therefore, the lifecycle of *P. obducens* did not begin in the true leaves of FLR and DOB. In the cotyledon of FLR, although the hyphae and haustoria could be observed occasionally, the extension of hyphae was greatly limited.

### Single-molecule real-time sequencing of transcriptomes and alternative splicing

To identify the genes controlling IDM resistance in *Impatiens*, we selected DOB (susceptible to IDM at the cotyledon stage, but resistant at the true leaf stage and thereafter) for full-length transcriptome characterization using the PacBio Iso-Seq protocol. In addition, SER (susceptible to IDM at the cotyledon, true leaf, and mature plant stages), FLR (resistant to IDM at the cotyledon, true leaf, and mature plant stages), and DOB were also selected for transcriptome profiling using the Illumina-based RNA-Seq methodology. To generate the full-length transcriptome of DOB, the RNA samples of cotyledon and true leaf tissues were pooled together for sequencing using the PacBio Sequel platform. A total of 716,913 raw polymerase reads (34.4 Gb) with an N50 length of 93,681 bp were generated on one SMRT cell (Supplementary Table S3). After running the bioinformatics pipeline, 464,436 circular consensus sequences (CCSs) were obtained, among which 418,648 were full-length non-chimeric (FLNC) reads containing the 5′ and 3′ primers and poly-A tails. After the clustering step, 36,954 high-quality (HQ) isoforms were generated. The minor low-quality (LQ) isoforms were excluded for further analysis. The HQ isoforms were further error-corrected using LoRDEC and trimmed RNA-Seq short reads described below. Subsequently, 36,954 error-corrected HQ isoforms were obtained with an N50 length of 3010 bp (Table 3).

**Table 3:** Summary statistics of Iso-Seq high-quality transcripts and RNA-Seq de novo assembly

	Iso-Seq		RNA-Seq					Reference set
	High-quality consensus isoforms	Collapsed isoforms	DOB-Trinity	FLR-Trinity	SER-Trinity	TGICL (combined)	CD-HIT-EST (combined)	
Total length (bp)	96,081,232	41,649,071	174,455,882	181,355,307	147,118,817	201,630,833	161,317,658	75,405,557
N50 (bp)	3010	2992	2112	2164	2242	2392	2277	2060
Number of sequences	36,954	16,752	118,919	120,416	95,837	120,538	100,049	48,758
Average length (bp)	2600	2486	1467	1506	1535	1673	1612	1547
Maximum length (bp)	11,302	11,302	16,637	18,052	19,009	19,009	19,009	16,726
Minimum length (bp)	196	237	300	300	300	300	300	298

*DOB* *I. hawkeri* Divine Orange Bronze Leaf, *FLR* *I. hawkeri* Florific Lavender, *SER* *I. walleriana* Super Elfin Red. The trimmed RNA-Seq reads belonging to the same cultivar were pooled for a de novo assembly using Trinity (--min_kmer_cov 2) (Grabherr et al.^75^). The three resulting assemblies were merged using TGICL v2.1 with default options (Pertea et al.^76^). Redundancy was removed using CD-HIT-EST (-c 0.95 -n 9 -T 0 -M 0 -r 1)

**Fig. 4:**
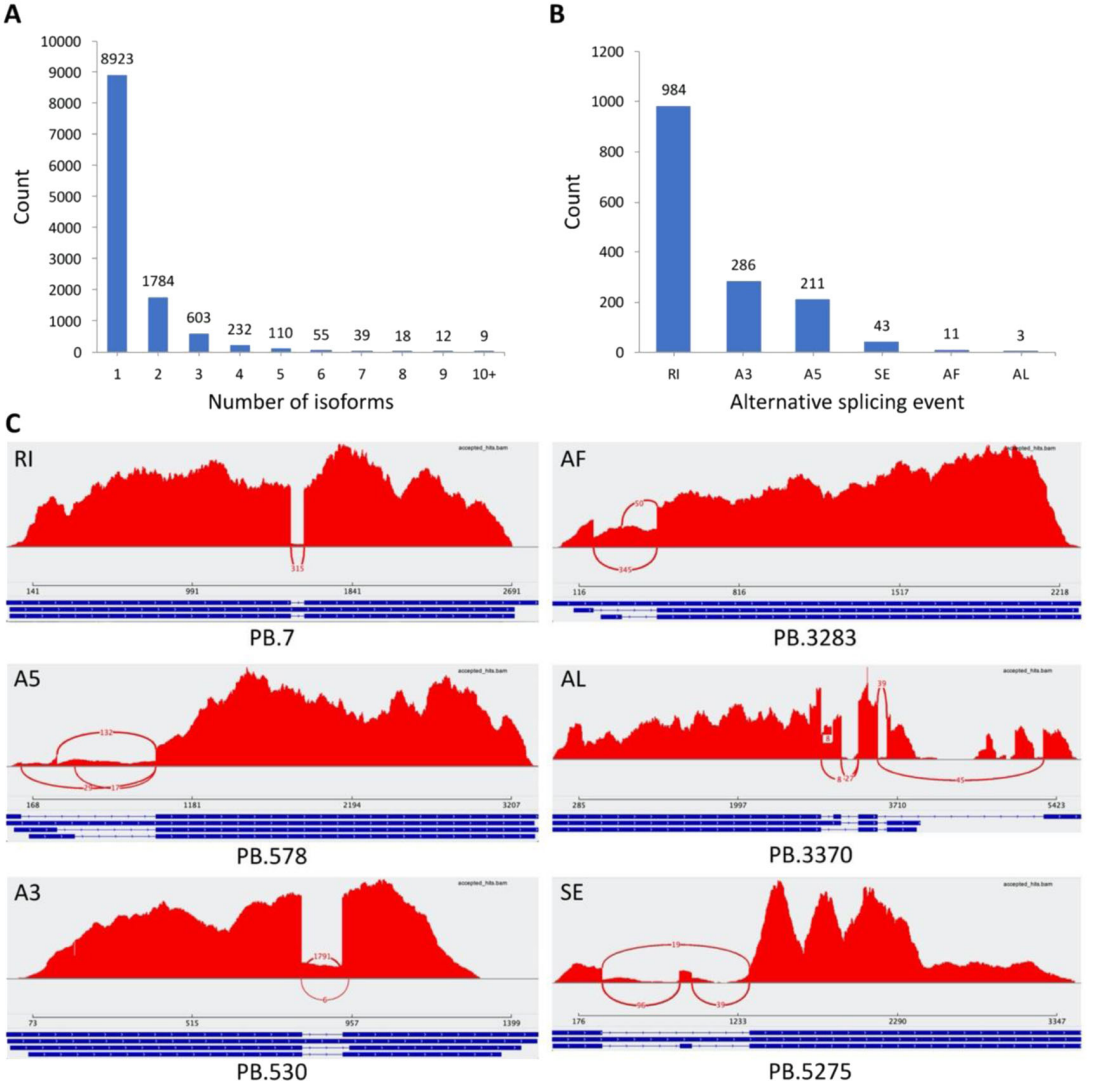
Isoform and alternative splicing (AS) analysis of *I. hawkeri* leaf transcripts using PacBio Iso-seq. **A** Distribution of isoform numbers based on UniTransModels. **B** Summary of AS events identified in the full-length transcriptome using UniTransModels as the reference. RI retained intron, A5 alternative 5′ splice-site, A3 alternative 3′ splice-site, SE skipping exon, AF alternative first exon, AL alternative last exon. **C** Sashimi plots showing examples of the six types of AS events. Red peaks indicate overage of Illumina short reads; curved red lines with numbers represent splicing junctions supported by this number of Illumina short reads. For isoforms located at the bottom, blue blocks represent exons and lines linking blocks represent introns

To discover alternative splicing (AS) events in *Impatiens*, the error-corrected and non-redundant (redundancy removed using CD-HIT-EST) HQ isoforms were partitioned into transcript families by the Coding GENome reconstruction Tool (Cogent) to reconstruct full-length unique transcript models (UniTransModels). A total of 11,763 full-length UniTransModels were obtained. Based on these UniTransModels, the HQ isoforms were further collapsed using Cupcake to obtain a set of 16,752 collapsed isoforms with an N50 length of 2992 bp (Table 3). Most of these UniTransModels (8,923, 75.7%) had one isoform, while 2862 (24.3%) UniTransModels had at least two isoforms (Fig. 4A, B). Based on these UniTransModels, there were six types of AS events observed, including retained intron (RI), alternative 5′ splice-site (A5), alternative 3′ splice-site (A3), skipping exon (SE), alternative first exon (AF), and alternative last exon (AL) (Fig. 4B). Among these AS events, RI type was the most predominant (984, 64.0%), followed by A3 (286, 18.6%) and A5 (13.7%). These three types of AS events accounted for >96% of detected events. By mapping the Illumina short reads to these UniTransModels, the reliability of detected AS events was confirmed (Fig. 4C).

### The first reference transcriptome of impatiens

For each of the three cultivars (SER, FLR, and DOB), the cotyledon and true leaf tissues were also subjected to Illumina short reads sequencing (RNA-Seq). A total of 18 samples (3 cultivars × 2 tissue types × 3 replicates = 18) were sequenced. An average of 14.5 million 150-bp cleaned read pairs were obtained for each sample (Supplementary Table S4). A de novo assembly was performed for each cultivar by pooling reads of the six samples and using Trinity. A total of 118,919, 120,416, and 95,837 contigs were obtained for DOB, FLR, and SER, with an N50 length of 2112, 2164, and 2242 bp, respectively (Table 3). The three RNA-Seq assemblies were merged using TGICL with redundancy removed using CD-HIT-EST, yielding 100,049 unique transcript sequences with an N50 length of 2277 bp. The unique transcript sequences obtained from RNA-Seq were mapped to Iso-Seq isoforms. For downstream functional annotation and investigation of gene expressions, a reference transcriptome for *Impatiens* was constructed by combining the longest collapsed isoforms from Iso-Seq and unmapped transcript sequences from RNA-Seq. Finally, a total of 48,758 reference transcript sequences with an N50 length of 2060 bp were obtained to represent the reference transcriptome of *Impatiens* (Table 3). To estimate the completeness of this reference transcriptome, we compared these sequences to the BUSCO embryophyta_odb9 dataset and obtained a completeness score of 85.2%.

**Fig. 5:**
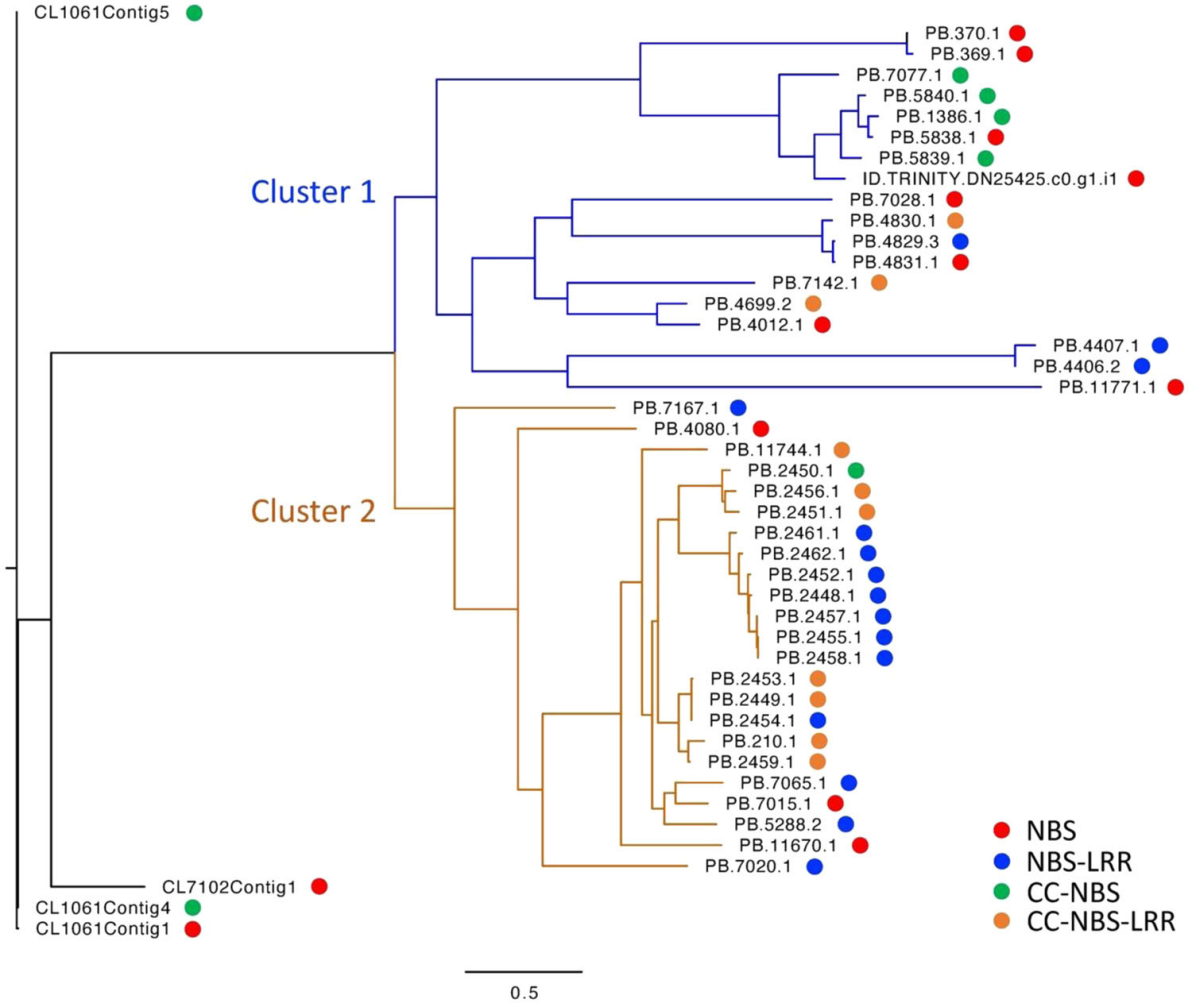
Phylogenetic tree of identified 45 NBS genes in the Impatiens leaf transcriptome. Two major clusters of NBS transcripts or genes were identified. The red, blue, green, and orange circles indicate the classification of the NBS genes, including NBS (red), NBS-LRR (blue), CC-NBS (green), and CC-NBS-LRR types (orange), respectively

For functional annotation, the reference transcriptome was compared to several major public databases. The majority of the sequences (36,978; 75.8%) had hits to the non-redundant protein (NR) database, followed by Swiss-Prot (29,731; 61.0%), and non-redundant nucleotide (NT) database (22,616; 46.4%) (Supplementary Table S5). A total of 22,699 (46.6%) transcripts were annotated with gene ontology (GO) terms, with an average of four GO terms per transcript. In addition, 11,537 (23.7%) sequences were assigned with Kyoto Encyclopedia of Genes and Genomes Ontology (KO) terms. By mining the reference transcriptome in the PlantTFDB v4.0 database, a small portion of sequences (1165; 2.4%) was predicted to encode transcription factors (TFs) and assigned to TF families (Supplementary Table S6). Among the 54 TF families, the most predominant was the bHLH family (122; 10.5%), followed by bZIP (77; 6.6%), and MYB-related (62; 5.3%). By running the TransDecoder pipeline, coding regions and protein sequences were successfully predicted for a total of 34,359 (70.5%) sequences, among which 27,515 (56.4%) sequences contained a complete open reading frame (ORF). Based on the predicted proteins out of the reference transcriptome, we identified 45 NBS-containing genes and 246 leucine-rich repeat receptor-like kinase (LRR-RLK) genes (Supplementary Table S7). Among these 45 predicted NBS genes, 33 (73.3%) contained a complete ORF. These NBS genes were further classified into four types, including NBS-LRR (15), NBS (13), coiled-coil (CC)-NBS-LRR (10), and CC-NBS (7). The TIR domain was not identified in these predicted NBS genes. A phylogenetic tree was constructed based on the NBS domain sequences, which revealed two major clusters of *Impatiens* NBS genes (Fig. 5).

### Identification of genes and *R*-genes potentially involved in downy mildew resistance

**Fig. 6:**
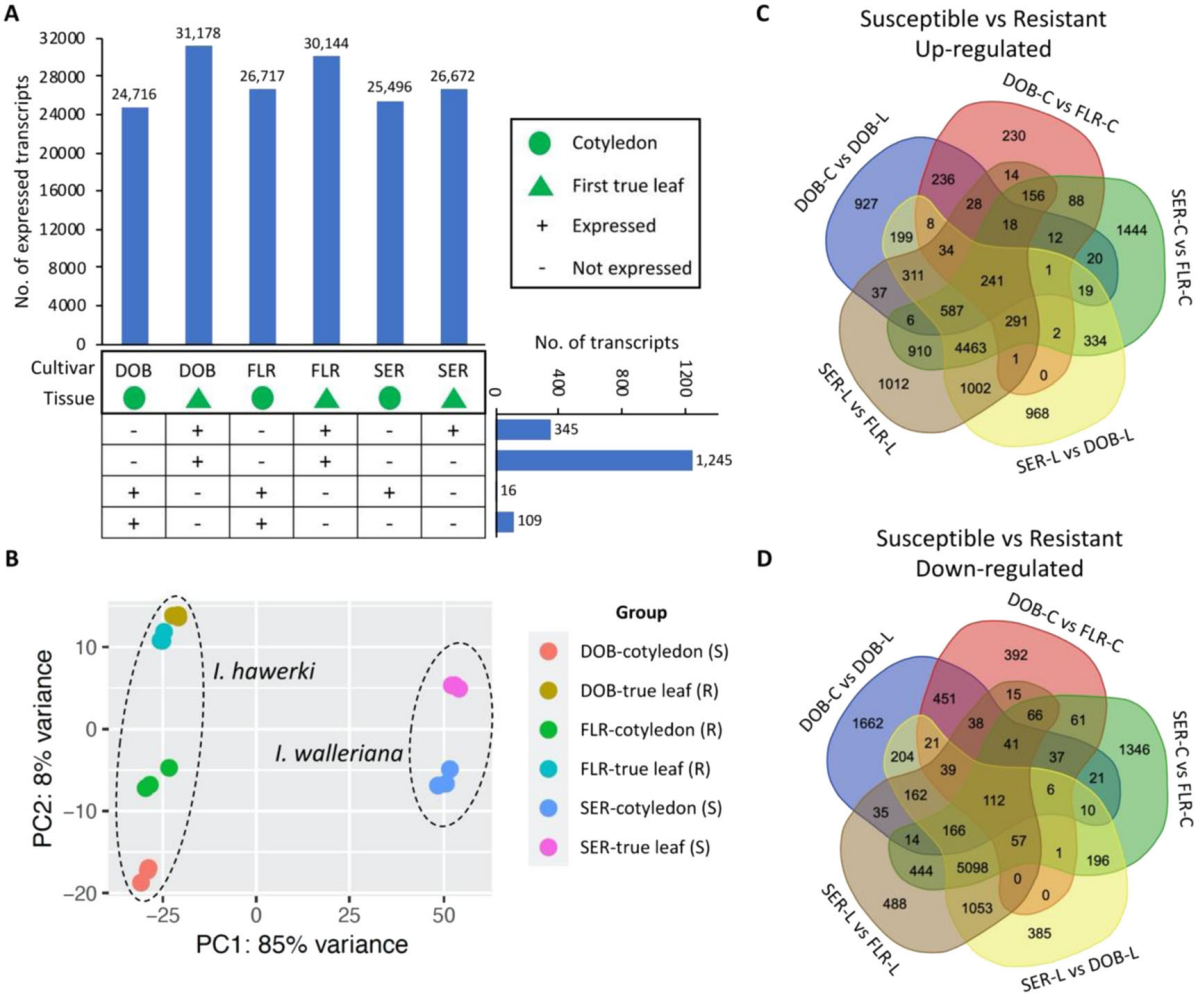
Gene expression profiles for impatiens cotyledons and true leaves, and differentially expressed genes between downy mildew-susceptible vs. -resistant samples. **A** Number of expressed transcripts (transcripts per million or TPM > 0.5) for each tissue type and each cultivar. DOB Divine Orange Bronze Leaf, SER Super Elfin Red, FLR Florific Lavender. Green circle represents cotyledon; green triangle represents true leaf. “+” indicates expressed; “−” indicates not expressed. The “No. of transcripts” (from top to bottom) corresponds to the transcripts that were only expressed in true leaves and not expressed in cotyledons for all three genotypes; transcripts that were only expressed in true leaves of *Impatiens hawkeri* and not expressed in cotyledons of *Impatiens hawkeri*, neither expressed in cotyledons nor true leaves of *Impatiens walleriana*; transcripts that were only expressed in cotyledons and not expressed in true leaves for all three genotypes; transcripts that were only expressed in cotyledons of *Impatiens hawkeri* and not expressed in true leaves of *Impatiens hawkeri*, neither expressed in cotyledons nor true leaves of *Impatiens walleriana*. **B** Principal component analysis of all genes using DESeq2 with normalized counts. Circles with different colors represent different groups of samples as shown in the figure. **C** Upregulated genes based on five downy mildew-susceptible vs. resistant sample comparisons. **D** Downregulated genes based on five susceptible and resistant sample comparisons

To identify impatiens *R*-genes, we first downloaded the 152 reference Pathogen Receptor Genes maintained at the PRGdb that have been cloned and well-characterized in other plant species. We also obtained 1678 proteins from NCBI and 37 Arabidopsis genes from the UniProt database based on their functionality in resistance to downy mildew. Through gene family analysis, we identified 683 impatiens genes (81 gene families) orthologous to the PRGdb reference *R*-genes or “downy mildew” associated genes (Supplementary Table S8). These impatiens orthologs and predicted NBS and LRR-RLK genes were prioritized for downstream evaluation of gene expressions in the five pairs of comparisons that were made possible by three cultivars (DOB, FLR, and SER) and two types of organs (cotyledon and true leaf) with different resistance or susceptibility to IDM (Fig. 6).

The clean reads from the 18 impatiens RNA-Seq samples were mapped to the reference transcriptome to investigate gene expression profiles of cotyledon and true leaf tissues of impatiens. By setting the transcripts per million (TPM) cutoff as 0.5 (at least one replicate) to be considered expressed, a range of 24,716–31,178 transcripts were expressed in the cotyledons and true leaves of these 18 samples (Fig. 6A and Supplementary Table S9). Apparently, a higher number of transcripts were expressed at the true leaf stage compared with the cotyledon stage for all three cultivars. Interestingly, DOB had the smallest number of expressed transcripts at the cotyledon stage but had the highest number of expressed transcripts at the true leaf stage. There were 345 transcripts only expressed at the true leaf stage for all three cultivars. A total of 1245 transcripts were only expressed at the true leaf stage for *I. hawkeri* samples, but not expressed in the *I. walleriana* sample. A much smaller number of transcripts were only expressed at the cotyledon stage. Through differential gene expression analysis using DESeq2, DOB had a much larger number of differentially expressed genes (DEGs) when transitioning from the cotyledon stage to the true leaf stage than the other two cultivars (Table 4 and Supplementary Table S10). Further principal component analysis (PCA) using DESeq2 also revealed that DOB had very different expression profiles between its cotyledons and true leaves. As shown in Fig. 6B, the expression profiles of true leaves were very similar between DOB and FLR, which belong to the same species. However, the cotyledon expression profiles of these two cultivars were separated apart. These unique features may correspond to DOB’s different responses to IDM at the cotyledon versus the true leaf stage compared with other *I. hawkeri* cultivars resistant to IDM at both stages. As expected, these two species were separated by the first PC, which explained 85% of the variance (PC1, 85%).

**Table 4:** Differentially expressed genes based on various comparisons

Comparison					Upregulated	Downregulated	Total
DOB cotyledon	S	vs	DOB-true leaf	R	2684	3019	5703
FLR cotyledon	R	vs	FLR-true leaf	R	1072	1091	2163
SER cotyledon	S	vs	SER-true leaf	S	977	873	1850
DOB cotyledon	S	vs	FLR cotyledon	R	1360	1337	2697
SER cotyledon	S	vs	FLR cotyledon	R	8592	7676	16,268
SER-true leaf	S	vs	DOB-true leaf	R	8461	7510	15,971
SER-true leaf	S	vs	FLR-true leaf	R	9111	7828	16,939

*DOB* *I*. *hawkeri* Divine Orange Bronze Leaf, *FLR* *I*. *hawkeri* Florific Lavender, *SER* I. *walleriana* Super Elfin Red. All comparisons have FDR < 0.05 and fold change ≥ 2. “S” indicates susceptibility to impatiens downy mildew; “R” indicates resistance to impatiens downy mildew

**Fig. 7:**
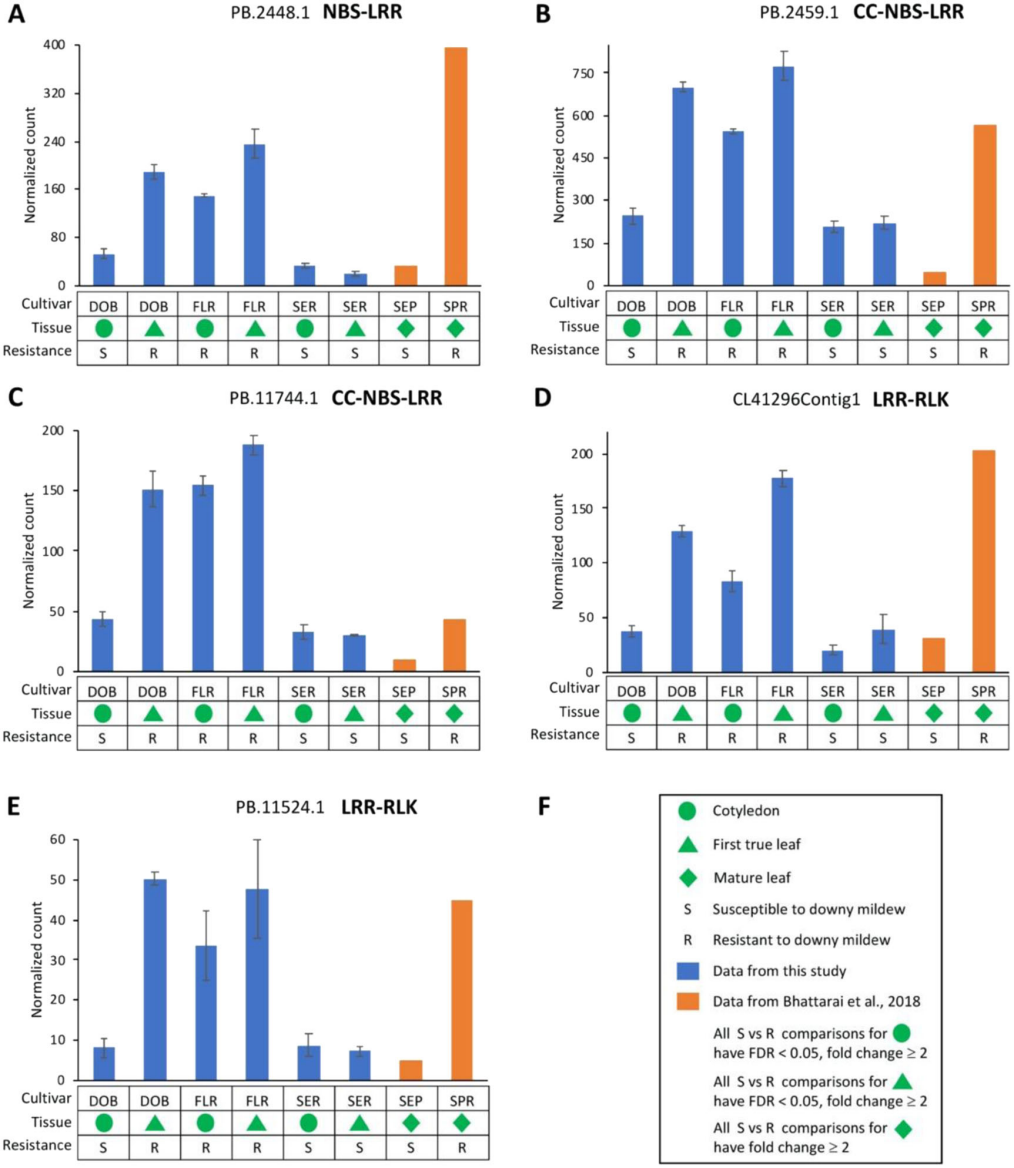
Differentially expressed genes associated with downy mildew resistance in impatiens. **A**–**C** Three nucleotide-binding site (NBS)-containing genes expressed at significantly higher levels in all downy mildew-resistant samples than in susceptible samples. **D**, **E** Two leucine-rich repeat receptor-like kinase (LRR-RLK) genes expressed at significantly higher levels in all resistant samples than in susceptible samples. **F** Symbols and signs in **A**–**E**. DOB Divine Orange Bronze Leaf, SER Super Elfin Red, FLR Florific Lavender, SEP Super Elfin Pink, SPR SunPatiens^®^ Compact Royal Magenta. The gene expressions in mature leaves of SEP and SPR were from a previously published, independent study^41^, and they were included for a comparison

**Fig. 8:**
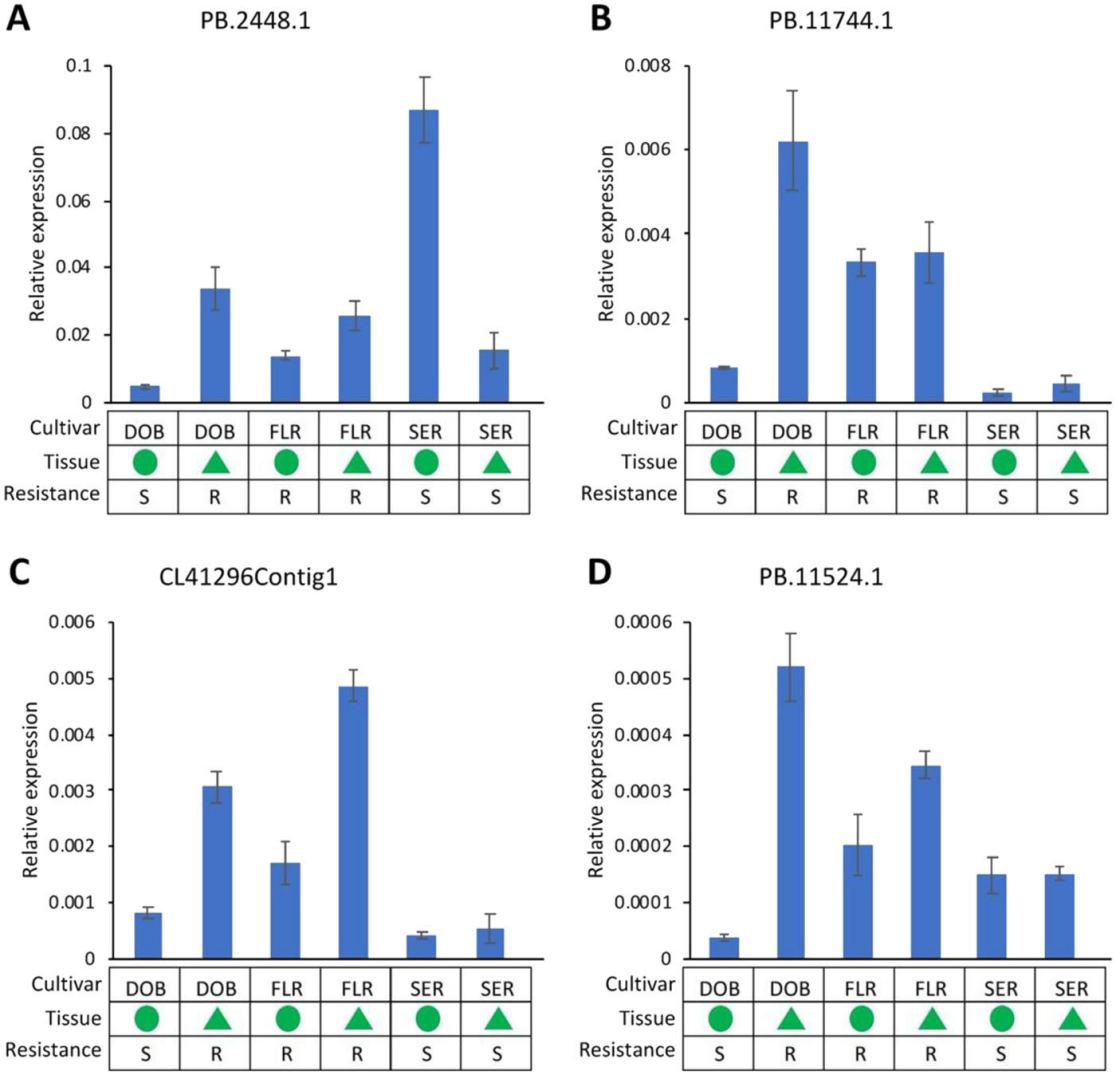
qRT-PCR validation of gene expressions in *I. hawkeri* and *I. walleriana* samples. The expression levels of selected candidate genes were normalized to *GAPDH*. **A**, **B** The relative expression of two nucleotide-binding site (NBS)-containing genes. **C**, **D** The relative expression of two leucine-rich repeat receptor-like kinase (LRR-RLK) genes. DOB Divine Orange Bronze Leaf, SER Super Elfin Red, FLR Florific Lavender

Given that DOB transitioned from IDM susceptibility (S) on cotyledons to IDM resistance (R) on true leaves, it was expected that the genes associated with resistance to IDM expressed differently in true leaves compared with cotyledons in DOB. Thus, candidate genes were first mined based on the following criteria: (1) differentially expressed (FDR < 0.05, fold change ≥ 2) for DOB cotyledon (S) vs DOB-true leaf (R); (2) within the same tissue type, for the genes upregulated in DOB-true leaf (R) compared with DOB cotyledon (S), we looked for those that were also expressed at higher levels (FDR < 0.05, fold change ≥2) in IDM-resistant cultivars than in susceptible cultivars; (3) similarly, for the genes downregulated in DOB-true leaf (R) compared with DOB cotyledon (S), we looked for those that were also expressed at lower levels (FDR < 0.05, fold change ≥2) in resistant cultivars than in susceptible cultivars (Table 4 and Supplementary Table S10). By applying these criteria, we identified 241 transcripts upregulated and 112 transcripts downregulated for all S vs R comparisons (Fig. 6C, D and Supplementary Table S11). Importantly and interestingly, three NBS genes orthologous to cloned and characterized *R*-genes and to those associated with DM resistance were among the 241 upregulated transcripts (Fig. 7A–C and Supplementary Table S8). These three NBS genes were expressed at significantly higher levels in all IDM-resistant samples compared with susceptible samples. For further verification, we also analyzed the gene expression data from another independent study on mature leaves (three replicates pooled for sequencing) of IDM-resistant and susceptible *Impatiens* cultivars^41^. We observed that these NBS genes were also expressed much higher in resistant *Impatiens* “SunPatiens^®^ Compact Royal Magenta” (SPR) than in susceptible sample SEP. The three NBS genes were orthologous to two genes (ACY69609.1/RGC203 and ADX86902.1) in common sunflower that have been associated with resistance to *Plasmopara halstedii*, the causal agent of sunflower DM^45,46^, and were also orthologous to two genes (*Rpi-blb1* and *RB*) conferring resistance to potato blight in *Solanum bulbocastanum*, a potato relative^47,48^. Considering this evidence, these NBS genes can be good candidates for future mining *Impatiens* genes conferring IDM resistance. In addition, we identified two LRR-RLK genes significantly upregulated in all IDM-resistant samples (Fig. 7D, E), which may also be candidates potentially associated with IDM resistance. The three candidate NBS genes and two candidate LRR-RLK genes were selected for qRT-PCR validation of gene expressions in *I. hawkeri* and *I. walleriana* samples. Several pairs of primers were designed for candidate gene PB.2459.1, but somehow all designed primers did not work properly for this candidate gene. The qRT-PCR results supported that the expression levels of PB.2448.1, PB. 11744.1, PB.11524.1, and CL41296Contig1 were much higher (fold change ≥2) in the resistant samples than in the susceptible samples from *I*. *hawkeri* (Fig. 8A–D). When *I. walleriana* samples were included in the qRT-PCR comparison, only PB.11744.1 (CC-NBS-LRR) and CL41296Contig1 (LRR-RLK) showed significantly higher expression levels in the resistant samples than in the susceptible samples. Since only a single reference gene (*GAPDH*) was used for normalization in these qRT-PCR analyses, caution might be warranted when comparing gene expression levels between *I*. *hawkeri* and *I*. *walleriana*.

## Discussion

As a worldwide challenge, DM has devastated many crops, including the ornamental crop garden impatiens, *I. walleriana*. *Impatiens hawkeri* has been reported to be generally resistant to IDM. However, to date, little research has been reported to develop disease screening or resistance phenotyping methodologies in *Impatiens* and to understand the IDM resistance in *I*. *hawkeri*. The lack of information on resistance phenotyping and resistance mechanism has severely hindered efforts to develop new IDM-resistant cultivars. Moreover, the interaction between *P. obducens* and *Impatiens* remains to be discovered. Therefore, to fill these gaps, we have established a rapid, efficient, and effective system to assess IDM susceptibility and resistance at very early growth stages (first and second true leaves as well as the cotyledon stage) and histologically characterized the pathogen development inside impatiens cotyledons and leaves. Using this newly developed method, we discovered that two cultivars (DL and FLR) possess strong IDM resistance starting at the earliest growth stage (the cotyledon stage) and that a number of other cultivars including DOB could make the dramatic transition within a short time and a very short distance, from susceptibility at the cotyledon stage to complete resistance at the first/second pair of true leaf stage. These cultivars and growth stages with different levels of resistance to IDM have created an excellent opportunity to investigate host–pathogen (impatiens—*P*. *obducens*) interactions and discover genes potentially involved in such a dramatic transition process. In this study, we grasped this opportunity and applied full-length transcriptome sequencing (Iso-Seq) and RNA-Seq to three cultivars (SER, DOB, and FLR) with contrasting phenotypes to IDM and made five pairs of transcriptome comparisons between IDM-resistant and susceptible cultivars and tissues. These comparisons enabled us to identify 241 transcripts upregulated in resistant cultivars and resistant tissues and three *R*-genes potentially involved in IDM resistance. Results and genomic resources from this study will help better understand IDM resistance in impatiens, develop molecular markers, implement genomics-assisted breeding, accelerate the development of new IDM-resistant cultivars, and provide genomic resources for cloning of IDM-resistance genes.

All *I. walleriana* cultivars tested in this study exhibited susceptibility to IDM throughout the entire plant developmental stages, from the cotyledon stage to mature, flowering plants. While *I. hawkeri* showed general resistance to IDM, many cultivars were susceptible to IDM at the cotyledon stage, indicating important influences of plant developmental stage (or tissue type) on IDM resistance or susceptibility. A similar phenomenon has been observed in certain other plant–pathogen interactions. For example, broccoli (*Brassica oleracea*) lines “PCB21.32” and “OL87123-2” were fully susceptible to DM (*Hyaloperonospora parasitica*) at the cotyledon stage but were resistant to the pathogen at 6-weeks old^49^. Therefore, for these plants, DM resistance cannot be predicted from cotyledon resistance. By contrast, cotyledons and true leaves in basil (*Ocimum basilicum*) exhibited similar DM responses, indicating that early inoculation could be used in DM resistance evaluation^50^. In our study, we observed different *I*. *hawkeri* cultivars had different responses to IDM at the cotyledon stage and even some of the *I*. *hawkeri* cultivars were highly susceptible to IDM at this early stage. Therefore, to prevent potential damage of IDM to these cultivars during young plant production, fungicide protection in the production facility is required at the cotyledon stage. As for breeding impatiens for IDM resistance, we recommend screening impatiens breeding populations beginning at the cotyledon stage so that newly developed resistant cultivars will have resistance through their entire plant developmental stages. Delayed IDM disease screening may result in new cultivars susceptible to this fast-acting and destructive disease at their early growth stages resulting in crop failure of large-scale young plant production. This very early stage disease screening should be invaluable to impatiens breeding: It identifies new breeding lines and ultimately new cultivars with lifetime-long resistance to IDM, and it should also greatly reduce the space, time, labor required, and costs associated with screening large numbers of impatiens breeding populations.

Hypersensitive response (HR) involves the rapid death of host cells that can limit the progress of infection. It is a plant resistance response that can be used to differentiate between resistant and susceptible plants. In different plants, varied HR symptoms have been reported when resistant hosts were infected by the DM pathogen. For instance, resistant *Arabidopsis* plants showed different HR symptoms against DM, such as flecking necrosis, necrosis, pitting necrosis, or trailing necrosis, depending on the strength and timing of the cell death response^51^. By contrast, susceptible accessions displayed heavy conidiophore sporulation without visible cell death. Compared with susceptible individuals, which did not show any visible reactions except sporulation, resistant grapevine showed HR with isolated necrosis, resulting in a significant reduction of pathogen expansion and disease symptoms^52^. In this study, “black spots” (and “black specks”) were observed on inoculated cotyledons of *I. hawkeri*. There was no simple linear correlation between disease incidence of IDM and black spot. This raised an interesting question as for what roles these black spots and specks may play in *I. hawkeri*’s resistance to IDM.

Although plants defend themselves against pathogens in different ways, successful host defenses disrupt the disease cycle primarily in the pre-penetration, penetration, or infection phase^53^. The host defense to DM in resistant plants has been studied in several plant species, such as Arabidopsis, lettuce, and grapevine^52,54–57^. Arabidopsis C24 appeared to develop HR upon infection with visible cell death and the elongation of hyphae branching out into the intercellular space was restricted^54^. In resistant transgenic lettuce, the growth process of pathogen *Bremia lactucae* was retarded and no sporophores were observed at any time points^55^. In these two examples, the lifecycle of DM pathogens in resistant plants was not completed. Whereas, in DM-resistant grapevines, *P. viticola* could complete its life cycle in leaf tissues, but its hyphal growth and sporangia formation were inhibited, resulting in no visible symptoms or sporulation^52,56,57^. The above three types of host defenses to DM all occurred in the infection phase. In comparison to above scenarios, we found that the resistance mechanism of *I. hawkeri* to *P. obducens* may be more similar to that of grapevines against *P. viticola*. Because in cotyledons of *I. hawkeri* FLR, the lifecycle of *P. obducens* could also complete occasionally, but hyphae growth, haustoria development, and sporulation were greatly restricted.

RNA-Seq has been a powerful approach to understanding the transcriptional regulations associated with the disease response to DM in many crops, such as grapevine^57,58^, spinach^40^, lima bean^59^, and pearl millet^60^. However, the short reads from RNA-Seq are usually insufficient to reconstruct an accurate transcriptome, especially for species without a publicly available reference genome. Our study has combined the strengths of the third-generation sequencing technology with a much longer read length and RNA-Seq in reconstructing the transcriptome for *Impatiens* spp. with limited genome and transcriptome resources. As revealed in this study, the N50 length of Iso-seq isoforms (2992 bp) is much longer than the final assembly from RNA-Seq (2277 bp). The full-length transcriptome not only provided full-length transcript sequences for genes but also provided insights into the AS events in *Impatiens* spp. In consistent with other crops such as cotton^61^, rice^62^, and Italian ryegrass^63^, the retained intron type contributed the majority of AS events in *Impatiens*. On the basis of Iso-seq isoforms and in combination with RNA-Seq assemblies, we constructed a reference transcriptome for *Impatiens* spp. with a BUSCO score of 85.2%. Since we only sequenced tissues collected from earlier developmental stages (cotyledon and true leaf stages), not all genes are expressed. This completeness is comparable to that of several reports in other plant species^64,65^.

Improving disease resistance is an important objective of impatiens breeding. Efficient screening of breeding populations to identify breeding lines with disease resistance is critical for a breeding effort toward such a goal. Development and application of molecular markers have been proposed as an effective approach to increasing the screening efficiency in impatiens disease-resistance breeding. However, few molecular markers have been reported for disease-resistance traits in impatiens, primarily due to the lack of genomic resources. The transcriptome sequences assembled in this study can play an important role in the future development of molecular markers for disease-resistance traits (and other traits) in impatiens. The 45 impatiens NBS and 246 LRR-RLK gene sequences identified in this study can be particularly valuable for this effort. It has been shown that NBS genes constitute the large family of *R*-genes conferring plants resistance to diverse bacterial, fungal, oomycete, and viral pathogens and nematodes, even insects in some cases^66^. LRR-RLK genes also play important roles in plant disease resistance, functioning as *R*-genes or as members of the plant defense signaling pathways. In other plants, NBS and LRR-RLK sequences often co-localize or are linked or associated with disease-resistance loci or QTLs, having allowed rapid development of new molecular markers^10^. Thus, these impatiens NBS and LRR-RLK sequences can serve as an excellent starting point in future efforts toward developing molecular markers for disease-resistance traits in impatiens. The full-length coding region sequences of these genes from the assembled reference impatiens genome can speed up the cloning and functional characterization of the identified impatiens disease-resistance genes.

One limitation of this study is that *P*. *obducens*-inoculated samples were not available to be included in transcriptome sequencing due to the lack of viable pathogen inoculum when this part of the study was initiated. Without *P*. *obducens*-inoculated samples in transcriptome sequencing, transcripts that were to be induced by pathogen infection could not be captured. To overcome this shortage, we made use of the unique impatiens genotype (DOB) that was discovered in this study and made comparisons between IDM-resistant and susceptible tissues and between IDM-resistant and susceptible cultivars. These comparisons enabled us to identify 241 and 112 transcripts upregulated and downregulated in IDM-resistant cultivars/tissues, respectively. These differentially expressed transcripts can be very valuable for further dissection of the interactions between impatiens and *P*. *obducens* at the molecular level. The three NBS and two LRR-RLK transcripts that were upregulated in the IDM-resistant cultivars/tissues may be of particular value because they were also expressed at higher levels in another IDM-resistant *I. hawkeri* cultivar in a previous paper^41^ and potentially involved in IDM resistance. Several approaches can be used in future experiments to test the roles of these transcripts in IDM resistance, including genetic segregation analysis, genetic mapping, gene expression analysis, genetic transformation and overexpression, and/or knockout with RNAi or gene editing^67^. The full-length coding region sequences of these transcripts can facilitate the initiation of all these important analyses.

For all three cultivars (DOB, FLR, and SER), a higher number of transcripts were expressed at the true leaf stage compared with the cotyledon stage, indicating that more genes are needed and induced as impatiens plants begin to grow and develop. However, it seems a smaller number of transcripts were newly induced in *I. walleriana* than in *I. hawkeri*. The *I. hawkeri* cultivar DOB seems a special case since the number of DEGs by comparing cotyledon and true leaf stage (5703) was at least twice of that in the other two cultivars (2163 or 1850). According to the PCA analysis, the high number of DEGs in DOB is less likely due to a distinct expression profile in true leaf, since DOB and FLR (both belonging to *I. hawkeri*) had very similar expression profiles in true leaf. Instead, it is more likely to be explained by the distinct expression profile of cotyledon of DOB, as DOB and FLR had relatively dissimilar expression profiles in the cotyledon. Moreover, the total number of transcripts expressed in cotyledons of DOB was only 24,716, the lowest among the three cultivars. Therefore, it is possible that some molecular or transcriptional regulations related to IDM resistance may be missing or undermined in cotyledons of DOB, but later came back to a level similar to FLR at the true leaf stage. We found these DEGs from DOB to be of particular interest since the genes associated with susceptibility (at the cotyledon stage) or resistance (at the true leaf stage) to IDM are likely among these DEGs. Subsequently, we further identified DEGs shared by all possible S vs R comparisons within the same tissue types, which could represent the transcriptional differences associated with susceptibility/resistance to IDM. The differential expression analysis combined with large-scale identification of NBS genes, LRR-RLK genes, and orthologs to public *R*-genes and genes associated with DM finally led to a few candidate genes, including three NBS genes.

Currently, >30 resistance genes against DM, designated as *Pl* genes, have been identified and extensively studied in sunflower^68,69^. NBS genes have played an important role. In sunflower, two types of DM resistance have been reported, including type I which restricts the pathogen growth in hypocotyls, and type II which allows the pathogen to reach hypocotyls and cotyledons^45^. The type II resistance (*Pl*_*14*_) was reported to be controlled by CC-NBS-LRR genes, while type I resistance (*Pl*_*ARG*_) likely controlled by TIR-NBS-LRR genes. Besides, the type II resistance gene (*Pl*_*14*_) was reported to be in close proximity to several clusters of non-TIR type NBS-LRR genes that appeared to be tandemly duplicated in the sunflower genome^46^. In comparison with the two types of resistance in sunflower, the resistance to IDM conferred by *I. hawkeri* DOB may be similar to the type II resistance. First, the cotyledons of DOB can be invaded by IDM. Second, the TIR domain was not identified in the NBS genes of impatiens, indicating most NBS genes of impatiens could be a non-TIR type. Moreover, the three NBS genes identified in this study were assigned to the same gene family with RGC203 (resistance type II) in sunflower, and two of these NBS genes are of a CC-NBS-LRR type. As a total of 20 impatiens NBS genes are assigned to this gene family, they may also belong to duplicated clusters, which needs further confirmation based on genome sequences. Future experiments can be designed to look at the temporal expressions of these candidate genes along with IDM infection and to investigate their functions. Since both susceptibility and resistance to IDM can be observed on the same plant at different growth stages, the cultivars like DOB would be an excellent plant material and model to further clarify the molecular mechanisms of IDM resistance and susceptibility in impatiens.

## Conclusion

In summary, our study investigated the resistance and susceptibility of *I. walleriana* and *I. hawkeri* cultivars to *P. obducens* at different plant growth stages. By artificial inoculation and histological characterization of pathogen development inside inoculated tissues, we established an effective early and rapid system to screen impatiens cultivars and breeding populations for IDM resistance and to study plant–pathogen interactions. Using this system, we discovered two cultivars with strong resistance to IDM from their cotyledon stage on and additional cultivars that expressed, at different growth stages, dramatically different levels of resistance to *P*. *obducens*. We took advantage of these newly discovered disease responses and further characterized the expression profiles of cotyledons and true leaves of *Impatiens*. Our study has provided a comprehensive data source for mining disease-resistance genes in *Impatiens*, including transcriptome-wide identified NBS genes, LRR-RLK genes, genes orthologous to public *R*-genes and downy mildew associated genes, and DEGs differentially regulated between resistant and susceptible cultivars and tissues. Our results have laid a solid foundation for further research to understand and improve DM resistance in impatiens and have good potential to be applied to other crops.

## Materials and methods

### *Impatiens walleriana* and *I*. *hawkeri* cultivars and seedlings

Sixteen cultivars of *I. walleriana* from Accent Premium, Xtreme, Super Elfin, and Balance series and 16 *I. hawkeri* from Florific and Divine series (Table 1 and Supplementary Table S1) were evaluated for their response to *P*. *obducens* infection at the cotyledon, first/second pair of true leaf, and mature plant stages. Seeds of these 32 cultivars were sown on 20-rowed germination trays (model P-SEED20; Landmark Plastic Co., Orlando, FL) filled with Fafard germination Mix (Conrad Fafard, Inc., Agawam, MA). The trays were covered with plastic lids to keep moisture in a growth room at temperatures between 22 and 25 °C and a photoperiod of 16 h light/8 h dark. Seedlings with cotyledons (about two weeks old for *I. walleriana* and three weeks old for *I. hawkeri*) were transferred, one plant per cell, into 128-cell trays (model TR128D; Speedling Inc., Sun City, FL) filled with the commercial potting mix Fafard 3B mix (Conrad Fafard, Inc.). Seedlings were grown in the DM-free greenhouse with the temperature controlled between 25 and 30 °C. A liquid fertilizer containing 20% (w/w) nitrogen, 20% (w/w) phosphate (P_2_O_5_), and 20% (w/w) potassium (K_2_O) (Southern Agricultural Insecticides Inc., Palmetto, FL) was applied to the seedlings at 75 ppm twice a week following the irrigation program. All seedlings used in different experiments were grown using this method and all experiments were conducted at the University of Florida’s Gulf Coast Research and Education Center (UF/GCREC) (lat. 27°45’36” N, long. 82°13’45” W; AHS Heat Zone 10; USD Cold Hardiness Zone 9 A) in Wimauma, FL, USA.

### Plant growth and field disease evaluation

On April 11, 2014 (47 days after seeds were sown), seedlings with four pairs of true leaves were transplanted into 72-cell trays (model TR72D; Speedling Inc., Sun City, FL) filled with the commercial potting mix Fafard 3B mix and kept in the greenhouse. The same liquid fertilizer was applied to the seedlings at 75 ppm once a day following the irrigation program. On June, 5 2014 (0 DAP), plants were transplanted on the 20-cm-high, 81-cm-wide raised ground beds of EauGallie fine sand covered with white-on-black plastic mulch in the experimental field of UF/GCREC. The overhead shade cloth was set up over the beds to create a partially shady environment (~40%). All cultivars were planted following a randomized complete block design with three blocks. For each cultivar, two biological replications in each block were grown 112.5 cm apart from each other. Drip irrigation with regular fertilizer and insecticide programs were followed. Plants were checked visually every 2 days for white sporulation on the abaxial side of leaves as an indication of IDM. Diseased leaves were sampled and observed under a bright-field microscope (BH-2) to confirm the pathogen identity.

### In vivo preservation of DM pathogen

*Plasmopora obducens* sporangia were obtained from *I. walleriana* Accent Premium Rose (APR) during a field trial in March 2015 and then used to inoculate susceptible *I. walleriana* APR stock plant maintained in an isolated growth room. First, identification of *P. obducens* causing DM was achieved by symptoms of plants, and the morphology of sporangiophores and sporangia described by Palmateer et al.^24^. A sporangia solution (1 × 10^5^ sporangia ml^−1^) was prepared as described by Pyne et al.^50^. Fresh sporulating leaves of APR were dipped into distilled water and gently agitated for 5 min. The *P*. *obducens* sporangia suspension was filtered through a 40-μm nylon mesh cell strainer (Thermo Fisher Scientific, Bridgewater, NJ) and then centrifuged at 3000×*g* for 10 min. This mesh cell strainer was used to remove debris and produce a cleaner sporangia suspension. Since *P. obducens* sporangia were ovoid and 12.7–25.0 × 10.0–17.7 µm in dimension^24,29^, they were expected to pass through the strainer easily. The supernatant was discarded, leaving the pellet re-suspended in 10 ml of distilled water. The sporangia density in the suspension was adjusted to a final density of 1 × 10^5^ sporangia ml^−1^ using a Reichert Bright-Line hemocytometer (Hausser Scientific, Horsham, PA) and a BH-2 microscope (Olympus America Inc., Melville, NY). The prepared sporangia suspension was finely sprayed onto the adaxial leaf surface of *I. walleriana* APR plants (60-days old). The inoculated plants were kept in closed plastic bags on a metal bench in the growth room with the air temperature maintained at 21 ± 1°C, light intensity of 160 µmol m^–2^ s^–1^, and 16-h light/8-h dark. The humidity inside the plastic bags was 100%, measured with hygrometers. After 7 days post inoculation (dpi), white downy growth was visualized on the abaxial leaf surface. Disease symptoms and the morphology of the sporangiophores and sporangia were compared to the control plants to verify the pathogen.

### Inoculating *I. walleriana* and *I. hawkeri* seedlings at three growth stages

Seedlings of 16 *I. walleriana* and 16 *I. hawkeri* cultivars were individually inoculated with *P. obducens* sporangia at the cotyledon stage or the first/second pair of true leaf stage in 128-cell trays. One droplet (~20 µL per droplet) of sporangia suspension (1 × 10^5^ sporangia mL^−1^) was added to the adaxial and abaxial sides of each cotyledon, respectively. To inoculate the first/second pair of true leaves, five droplets were applied onto the adaxial and abaxial sides of each leaf, respectively. Inoculated seedlings were immediately enclosed inside a polythene bag for 20 days. In the control treatment (non-inoculated), the cotyledons and first/second pair of true leaves were mock-inoculated with the same numbers of droplets of distilled water, and these seedlings were kept in a separate growth room with the same growing conditions. Inoculated seedlings were evaluated for IDM disease symptoms at 10 and 20 dpi. The disease incidence index was defined as mean ratings for downy mildew incidence using a binary scale, in which 0 equaled no visible sporulation and 1 equaled visible sporulation on the abaxial side of the cotyledon. A randomized complete block design with three replicates was used as the experimental design. For each replicate, 12 or 16 cotyledons or leaves were sampled per cultivar. Data analysis was performed in SAS version 8.1 (SAS Institute Inc., Cary, NC).

### Observation of pathogen development in inoculated cotyledons and true leaves

Based on in vivo inoculation results, *I. walleriana* SER, *I. hawkeri* DOB, and FLR were selected for microscopic observation. Infected cotyledon and true leaf segments (~5 × 10 mm) were collected, washed with autoclaved distilled water three times, and placed on 1% autoclaved water agar in plastic disposable Petri dishes (9.5 cm in diameter; 20 mL per dish). Cotyledon and leaf segments were inoculated with one 10-µL droplet at 1 × 10^5^ sporangia mL^−1^ on the adaxial surface. The inoculated cotyledon and true leaf segments were incubated for 24 h under the above-described conditions. Thereafter, the sporangia suspension droplets were blotted with autoclaved filter papers. The cotyledon and true leaf segments were kept on the water agar with the abaxial surface up to observe disease symptoms.

The development of *P. obducens* in impatiens cotyledons or true leaves was examined by microscopic observation of trypan blue-stained impatiens tissues. Five inoculated cotyledon or leaf segments per treatment were removed from the Petri dishes at 1, 2, 3, 4, 5, and 6 dpi and fixed by soaking them in 5 mL of the clearing solution A (acetic acid:ethanol = 1:3, v/v) in a 50-mL tube (one or two segments per tube). Tubes were shaken at a low speed (80 rpm) overnight. Subsequently, the clearing solution A was removed and replaced with 5 mL of the clearing solution B (acetic acid:ethanol:glycerol = 1:5:1, v/v/v). The tissue samples were shaken for at least 3 h and then treated with 5 mL of 0.01 % trypan blue (Sigma-Aldrich) staining solution (trypan blue:lactic acid:phenol:distilled water = 0.003:1:1:1, w/v/v/v). Impatiens tissue samples were stained overnight on a shaker at a low speed (80 rpm). Stained cotyledon or leaf tissues were rinsed with a small amount of autoclaved 60% glycerol to remove the staining solution, immersed in 5 mL of autoclaved 60% glycerol, and shaken at 80 rpm for at least 2 h. Finally, the well-stained impatiens tissue samples were placed on a clean glass slide in a drop of 60% glycerol, covered with a coverslip, and observed under a microscope (BX41) equipped with an Olympus Q-color 5 camera (Olympus America Inc., Melville, NY).

### Determination of sporangia densities on inoculated cotyledons and true leaves

Small pieces (5 × 5 mm) of tissue from inoculated cotyledon and leaf segments were cut and immersed in 200 µL distilled water amended with Tween 20 (0.05%; Sigma-Aldrich, St. Louis, MO) in a 1.5-mL microcentrifuge tube. The tubes were vortexed on a mini-shaker (Vortex-Genie; Fisher Scientific, Waltham, MA) for 5–10 s to dislodge the sporangia from cotyledon or leaf surfaces. Sporangia in the suspension were counted using a hemocytometer under a bright-field microscope (BH-2). Sporangia counts were converted into sporangia densities (total number of sporangia/area of the cotyledon or leaf segment sampled). Sporangia counting was performed every two days from 4 dpi to 10 dpi. For each time point, eight pieces of cotyledon or leaf segments were sampled. This experiment was repeated three times.

### Library preparation and sequencing

To investigate the normal transcriptome profiles of IDM-resistant and susceptible cultivars at cotyledon and true leaf stages, three representative cultivars were selected, including DOB (susceptible to IDM at the cotyledon stage; resistant to IDM at the true leaf stage and thereafter), SER (susceptible to IDM at all stages), and FLR (resistant to IDM at all stages). Seeds were planted on a commercial potting mix in containers and germinated in a greenhouse facility at UF/GCREC, USA. Cotyledons and first true leaves were collected at the cotyledon stage and true leaf stage, respectively, without *P. obducens* inoculation. For each tissue type/cultivar, samples were collected from three biological replicates. Collected samples were immediately frozen in liquid nitrogen for RNA extraction. RNA samples were extracted using RNeasy Plus Mini kit (Qiagen, CA, USA). RNA quality and quantity were evaluated using Qubit fluorometer 2.0 (Thermo Fisher Scientific, Waltham, USA) and Agilent 2100 Bioanalyzer (Agilent Technologies, CA, USA), respectively. The RNA samples of cotyledons and true leaves of DOB were pooled in equal amounts for PacBio Iso-seq. The SMARTer PCR cDNA Synthesis Kit (Clontech, CA, USA) was used for full-length cDNA synthesis. Two size bins (<3 Kb and >3 Kb) were used for cDNA fraction and Iso-Seq library construction, which was sequenced on one SMRT cell of the PacBio Sequel system (PacBio, CA, USA) at the Interdisciplinary Center for Biotechnology Research, University of Florida, Gainesville, FL, USA. In addition, the RNA samples from the above three cultivars were sent to the University of California Davis Genome Center for Illumina HiSeq4000 and NovaSeq6000 sequencing (150 bp paired-end reads).

### Iso-Seq and RNA-Seq data analysis

The Iso-Seq raw data were processed following the PacBio Iso-Seq pipeline using SMRT Link v8.0 and Iso-Seq3 (https://github.com/PacificBiosciences/IsoSeq_SA3nUP). Only high-quality (HQ) consensus sequences were used for further analysis. The trimmed Illumina short reads below were used to correct errors in the HQ consensus sequences using LoRDEC^70^. The redundancy was removed using CD-HIT-EST^71^ (-c 0.99 -n 10 -T 0 -M 0 -r 1). Cogent v2.1 was used to reconstruct the unique transcript models (UniTransModels) (https://github.com/Magdoll/Cogent). The error-corrected and non-redundant HQ consensus sequences were mapped to the UniTransModels using GMAP and further collapsed using Cupcake (https://github.com/Magdoll/cDNA_Cupcake). The alternative splicing (AS) events were identified using SUPPA^72^ with default settings. For visualization of AS events, sashimi plots were generated using the Integrative Genomics Viewer (IGV). The bam file was obtained by aligning the Illumina short reads of DOB to the UniTransModels using Tophat2^73^.

The raw Illumina reads (HiSeq and NovaSeq) were trimmed using Trimmomatic^74^. The trimmed reads belonging to the same cultivar were pooled for a de novo assembly using Trinity^75^, respectively (--min_kmer_cov 2). The three resulting assemblies were merged using TGICL v2.1 with default options^76^. Redundancy was removed using CD-HIT-EST (-c 0.95 -n 9 -T 0 -M 0 -r 1). To construct a reference transcriptome for downstream annotation and gene expression analyses, the final transcripts from RNA-Seq were mapped to the Iso-seq isoforms using BWA-mem^77^. The longest isoforms from Iso-seq and unmapped transcripts from RNA-Seq assembly were combined to represent the reference transcriptome of *Impatiens* spp. To assess the completeness of the reference transcriptome, it was compared to the BUSCO OrthoDB9 embryophyta dataset.

### Functional annotation and prediction of coding sequences

The reference transcriptome was compared to the non-redundant protein (nr), non-redundant nucleotide (nt) databases from NCBI (https://www.ncbi.nlm.nih.gov/), Swiss-Prot database (https://www.uniprot.org/), and Kyoto Encyclopedia of Genes and Genomes (KEGG) database (http://www.genome.jp/kaas-bin/kaas_main) using Blast (*E*-value ≤1e-05). Gene ontology (GO) terms were assigned using Blast2Go^78^ (-v -annot -dat -img -ips ipsr -annex -goslim). The Plant Transcription Factor Database (PlantTFDB) v4.0 (http://planttfdb.cbi.pku.edu.cn/prediction.php) was used to predict transcription factors (TFs). The coding sequences (CDS) and protein sequences were predicted following the TransDecoder pipeline (https://github.com/TransDecoder/TransDecoder) integrating the Blast (Swiss-Prot) and Pfam search results.

The NBS-containing genes were predicted by searching (hmmsearch) the predicted protein sequences using the hidden Markov model (HMM) profile of the NBS (PF00931)^79^ under *E*-value 1 × 10^−4^. PfamScan and NCBI Conserved Domain Search were used for confirmation of the NBS domain. The classification of NBS genes based on TIR, LRR, and CC domains was performed using NCBI Conserved Domains tool and Marcoil^80^ (probability > 90%). The NBS domain sequences were retrieved to construct a phylogenetic tree using RAxML under ‘PROTGAMMAJTTF’ model with 1000 bootstraps^81^. The identification of LRR-RLK genes followed the same method described previously^82^.

### Comprehensive search for downy mildew associated genes and gene family analysis

To collect publicly available plant proteins associated with DM, the keyword “downy mildew” was first searched at NCBI and 1678 proteins were obtained. The keyword was also searched at UniProt and 37 proteins from Arabidopsis were obtained. In addition, the 152 reference Pathogen Receptor Genes maintained at PRGdb (http://prgdb.org/prgdb/) were also included. To identify *Impatiens* orthologs, gene family analysis was performed for the above-collected proteins and predicted proteins from *Impatiens* using All-Against-All Blast (*E*-value 1 × 10^−5^) and OrthoMCL^83^.

### Differential expression analysis

The clean reads for each replicate were aligned to the reference transcriptome using BWA-mem. Only uniquely mapped reads were considered for further analysis. Read counts were obtained using HTSeq^84^. Differentially expressed genes (DEGs) were identified using DESeq2^85^ under the cutoff of false discovery rate (FDR) < 0.05 and fold change ≥2. The transcripts per million (TPM) values were calculated using TPMCalculator^86^.

### qRT-PCR validation

Two NBS genes and two LRR-RLK genes were selected for validation of gene expression in *I. hawkeri* samples using qRT-PCR. Primers were designed using BatchPrimer3 v1.0 (http://probes.pw.usda.gov/batchprimer3/). The RNA samples of cotyledon and true leaf tissues for DOB and FLR were used for cDNA synthesis with the SuperScript^®^ III First-Strand Synthesis System for RT-PCR kit (Invitrogen, CA, USA). qRT-PCR was carried out with three biological replicates and each containing two technical replicates for each tissue type using the Power SYBR^®^ Green PCR Master Mix kit (Applied Biosystems, USA). The cDNA levels of selected genes were normalized to the reference gene *GAPDH*.

## Supplementary information

Supplementary Tables

## Data Availability

The sequencing data and transcript assemblies generated from this study have been deposited at GenBank under SRA accession number PRJNA700505.

## References

[1] Thines, M., Voglmayr, H. & Göker, M. in *Oomycete Genetics and Genomics: Diversity, Interactions, and Research Tools*. (eds Lamour, K. & Kamoun, S.) 47–75 (John Wiley & Sons, 2009).

[2] Delmas CE (2016). Adaptation of a plant pathogen to partial host resistance: selection for greater aggressiveness in grapevine downy mildew. Evol. Appl..

[3] Zhang N, Lindhout P, Niks R, Jeuken M (2009). Genetic dissection of *Lactuca saligna* nonhost resistance to downy mildew at various lettuce developmental stages. Plant Pathol..

[4] Meena P, Thomas L, Singh D (2016). Assessment of yield losses in *Brassica juncea* due to downy mildew (*Hyaloperonospora brassicae*). J. Oilseed Brassica.

[5] Chelpuri D (2019). Mapping quantitative trait loci (QTLs) associated with resistance to major pathotype-isolates of pearl millet downy mildew pathogen. Eur. J. Plant Pathol..

[6] Lebeda, A., Křístková, E., Sedláková, B. & Widrlechner, M. P. Recent advances in cucurbit downy mildew research and their contribution to the development of integrated protection of cucurbits. *Ecol. Evol. Org. Biol. Proc.* **3**. https://lib.dr.iastate.edu/eeob_ag_conf/3 (2019).

[7] Delmas CE (2017). Soft selective sweeps in fungicide resistance evolution: recurrent mutations without fitness costs in grapevine downy mildew. Mol. Ecol..

[8] Callan, B. & Carris, L. in *Biodiversity of Fungi: Inventory and Monitoring Methods*. (eds Mueller, G. M., Bills, G. F. & Foster, M. S.) 105–126 (Elsevier Academic Press, 2004).

[9] Pecrix Y, Penouilh-Suzette C, Muños S, Vear F, Godiard L (2018). Ten broad spectrum resistances to downy mildew physically mapped on the sunflower genome. Front. Plant Sci..

[10] Parra L (2016). Rationalization of genes for resistance to *Bremia lactucae* in lettuce. Euphytica.

[11] Vezzulli S (2019). The *Rpv3-3* haplotype and stilbenoid induction mediate downy mildew resistance in a grapevine interspecific population. Front. Plant Sci..

[12] Radwan O, Bouzidi MF, Nicolas P, Mouzeyar S (2004). Development of PCR markers for the *Pl5/Pl8* locus for resistance to *Plasmopara halstedii* in sunflower, *Helianthus annuus* L. from complete CC-NBS-LRR sequences. Theor. Appl. Genet..

[13] Michelmore R, Wong J (2008). Classical and molecular genetics of *Bremia lactucae*, cause of lettuce downy mildew. Eur. J. Plant Pathol..

[14] Hermanns M, Slusarenko AJ, Schlaich NL (2003). Organ-specificity in a plant disease is determined independently of *R* gene signaling. Mol. Plant-Microbe Interact.

[15] She H (2018). Fine mapping and candidate gene screening of the downy mildew resistance gene *RPF1* in Spinach. Theor. Appl. Genet..

[16] Morgan, R. J. (ed.) *Impatiens: the Vibrant World of Busy Lizzies, Balsams, and Touch-me-nots* (Timber Press, 2007).

[17] Lim, T. (ed.) *Edible Medicinal and Non-medicinal Plants* (Springer, 2014).

[18] Uchneat, M. S. (ed.) *Flower Breeding and Genetics* (Springer, 2007).

[19] US Department of Agriculture, National Agricultural Statistics Service. https://www.nass.usda.gov (2019).

[20] Wang W, He Y, Cao Z, Deng Z (2018). Induction of tetraploids in impatiens (*Impatiens walleriana*) and characterization of their changes in morphology and resistance to downy mildew. HortScience.

[21] Vajna L (2011). First report of *Plasmopara obducens* on impatiens (*Impatiens walleriana*) in Hungary. New Dis. Rep.

[22] Cunnington J, Aldaoud R, Loh M, Washington W, Irvine G (2008). First record of *Plasmopara obducens* (downy mildew) on impatiens in Australia. Plant Pathol..

[23] Crouch J, Ko M, McKemy J (2014). First report of impatiens downy mildew outbreaks caused by *Plasmopara obducens* throughout the Hawai’ian islands. Plant Dis..

[24] Palmateer A, Lopez P, Seijo T, Peres N (2013). Severe outbreak of downy mildew caused by *Plasmopara obducens* on *Impatiens walleriana* in Florida. Plant Dis..

[25] US Department of Agriculture, National Agricultural Statistics Service. https://www.nass.usda.gov (2016).

[26] Conner K, Olive J, Hagan A, Zhang L, Bloodworth M (2014). First report of impatiens downy mildew caused by *Plasmopara obducens* in Alabama. Plant Dis..

[27] Warfield CY (2012). Downy mildew of impatiens. GrowerTalks.

[28] Hansen, M. A., Bush, E. A., Latimer, J. G. & Hong, C. *Impatiens Downy Mildew* (Virginia Coop Extension Virgia State Univ., 2013).

[29] Satou M, Sugawara K, Nagashima S, Tsukamoto T, Matsushita Y (2013). Downy mildew of busy lizzie caused by *Plasmopara obducens* in Japan. J. Gen. Plant Pathol..

[30] Jones, D. & O’Neill, T. Impatiens downy mildew. *East Malling, UK: Horticultural Development Council FactSheet* **5**, 8 (2004).

[31] Choi YJ, Han JG, Park MJ, Shin HD (2009). Downy mildew of *Impatiens balsamina* and *I. walleriana* in Korea. Plant Pathol. J..

[32] Keach JE, Bridgen M (2016). Towards improvement of *Impatiens*. Acta Hortic..

[33] Eskandari, F. & Shishkoff, N. Systemic infection of Impatiens balsamina through inoculation of roots with vegetative sporangia of the Impatiens downy mildew (*Plasmopara obducens*). *Phytopathol.* **107**, S4.3 (2017).

[34] Suarez S, Palmateer A (2014). Overview of impatiens downy mildew in Florida. Phytopathol.

[35] Suarez S, Lopez P, Chase A, Palmateer A (2016). Preventative fungicide applications in production and their impact on residual efficacy against impatiens downy mildew in the landscape. Phytopathol..

[36] Gessler C, Pertot I, Perazzolli M (2011). *Plasmopara viticola*: a review of knowledge on downy mildew of grapevine and effective disease management. Phytopathol. Mediterr..

[37] Stassen JH (2012). Effector identification in the lettuce downy mildew *Bremia lactucae* by massively parallel transcriptome sequencing. Mol. Plant Pathol..

[38] Li X (2015). Comparative transcriptome analysis reveals defense-related genes and pathways against downy mildew in *Vitis amurensis* grapevine. Plant Physiol. Biochem..

[39] Mestre P (2016). Comparative analysis of expressed CRN and RXLR effectors from two *Plasmopara* species causing grapevine and sunflower downy mildew. Plant Pathol..

[40] Kandel SL (2020). Transcriptional analyses of differential cultivars during resistant and susceptible interactions with *Peronospora effusa*, the causal agent of spinach downy mildew. Sci. Rep..

[41] Bhattarai K, Wang W, Cao Z, Deng Z (2018). Comparative analysis of impatiens leaf transcriptomes reveal candidate genes for resistance to downy mildew caused by *Plasmopara obducens*. Int. J. Mol. Sci..

[42] Suarez, S., Naveed, Z. A. & Ali, G. Transcriptional profiling of Impatiens walleriana genes through different stages of downy mildew infection reveals novel genes involved in disease susceptibility. Preprint at https://www.biorxiv.org/content/10.1101/622480v2 (2019).

[43] Rhoads A, Au KF (2015). PacBio sequencing and its applications. Genomics Proteomics Bioinformatics.

[44] Florida Automated Weather Network. https://fawn.ifas.ufl.edu (2015).

[45] Radwan O, Bouzidi MF, Mouzeyar S (2011). Molecular characterization of two types of resistance in sunflower to *Plasmopara halstedii*, the causal agent of downy mildew. Phytopathol..

[46] Bachlava E (2011). Downy mildew (*Pl*_8_ and *Pl*_14_) and rust (*R*_Adv_) resistance genes reside in close proximity to tandemly duplicated clusters of non-TIR-like NBS-LRR-encoding genes on sunflower chromosomes 1 and 13. Theor. Appl. Genet..

[47] Song J (2003). Gene *RB* cloned from *Solanum bulbocastanum* confers broad spectrum resistance to potato late blight. Proc. Natl Acad. Sci. USA.

[48] Van Der Vossen E (2003). An ancient *R* gene from the wild potato species Solanum bulbocastanum confers broad-spectrum resistance to *Phytophthora infestans* in cultivated potato and tomato. Plant J..

[49] Coelho PS, Valério L, Monteiro AA (2009). Leaf position, leaf age and plant age affect the expression of downy mildew resistance in *Brassica oleracea*. Eur. J. Plant Pathol..

[50] Pyne RM, Koroch AR, Wyenandt CA, Simon JE (2014). A rapid screening approach to identify resistance to basil downy mildew (*Peronospora belbahrii*). HortScience.

[51] Coates ME, Beynon JL (2010). *Hyaloperonospora arabidopsidis* as a pathogen model. Ann. Rev. Phytopathol..

[52] Bellin D (2009). Resistance to *Plasmopara viticola* in grapevine ‘Bianca’is controlled by a major dominant gene causing localised necrosis at the infection site. Theor. Appl. Genet..

[53] Trigiano, R. N. in *Plant Pathology Concepts and Laboratory Exercises* (eds Ownley, B. H. & Trigiano, R. N.) 349–356 (CRC Press, 2007).

[54] Lapin D, Meyer RC, Takahashi H, Bechtold U, Van den Ackerveken G (2012). Broad-spectrum resistance of Arabidopsis C24 to downy mildew is mediated by different combinations of isolate-specific loci. New Phytol..

[55] Govindarajulu M, Epstein L, Wroblewski T, Michelmore RW (2015). Host‐induced gene silencing inhibits the biotrophic pathogen causing downy mildew of lettuce. Plant Biotechnol. J..

[56] Diez-Navajas A, Wiedemann-Merdinoglu S, Greif C, Merdinoglu D (2008). Nonhost versus host resistance to the grapevine downy mildew, *Plasmopara viticola*, studied at the tissue level. Phytopathol..

[57] Yin X (2017). Pathogen development and host responses to *Plasmopara viticola* in resistant and susceptible grapevines: an ultrastructural study. Hortic. Res..

[58] Perazzolli M (2012). Downy mildew resistance induced by *Trichoderma harzianum* T39 in susceptible grapevines partially mimics transcriptional changes of resistant genotypes. BMC Genomics.

[59] Kunjeti SG (2012). RNA‐Seq reveals infection‐related global gene changes in *Phytophthora phaseoli*, the causal agent of lima bean downy mildew. Mol. Plant Pathol..

[60] Kulkarni KS (2016). De novo transcriptome sequencing to dissect candidate genes associated with pearl millet-downy mildew (*Sclerospora graminicola* Sacc.) interaction. Front. Plant Sci..

[61] Wang M (2018). A global survey of alternative splicing in allopolyploid cotton: landscape, complexity and regulation. New Phytol..

[62] Zhang G (2019). PacBio full‐length cDNA sequencing integrated with RNA‐seq reads drastically improves the discovery of splicing transcripts in rice. Plant J..

[63] Hu Z (2020). Full-length transcriptome assembly of Italian ryegrass root integrated with RNA-Seq to identify genes in response to plant cadmium stress. Int. J. Mol. Sci..

[64] Feng S, Xu M, Liu F, Cui C, Zhou B (2019). Reconstruction of the full-length transcriptome atlas using PacBio Iso-Seq provides insight into the alternative splicing in *Gossypium australe*. BMC Plant Biol..

[65] Kuang X, Sun S, Wei J, Li Y, Sun C (2019). Iso-Seq analysis of the *Taxus cuspidata* transcriptome reveals the complexity of Taxol biosynthesis. BMC Plant Biol..

[66] Tan X (2007). Global expression analysis of nucleotide binding site-leucine rich repeat-encoding and related genes in Arabidopsis. BMC Plant Biol..

[67] Li MY (2020). CRISPR/Cas9-mediated *VvPR4b* editing decreases downy mildew resistance in grapevine (*Vitis vinifera* L.). Hortic. Res..

[68] Qi L, Ma G, Li X, Seiler G (2019). Diversification of the downy mildew resistance gene pool by introgression of a new gene, *Pl*_35_, from wild *Helianthus argophyllus* into oilseed and confection sunflowers (*Helianthus annuus* L.). Theor. Appl. Genet..

[69] Ma G (2019). Molecular dissection of resistance gene cluster and candidate gene identification of *Pl*_17_ and *Pl*_19_ in sunflower by whole-genome resequencing. Sci. Rep..

[70] Salmela L, Rivals E (2014). LoRDEC: accurate and efficient long read error correction. Bioinformatics.

[71] Li W, Godzik A (2006). Cd-hit: a fast program for clustering and comparing large sets of protein or nucleotide sequences. Bioinformatics.

[72] Alamancos GP, Pagès A, Trincado JL, Bellora N, Eyras E (2015). Leveraging transcript quantification for fast computation of alternative splicing profiles. RNA.

[73] Kim D (2013). TopHat2: accurate alignment of transcriptomes in the presence of insertions, deletions and gene fusions. Genome Biol..

[74] Bolger AM, Lohse M, Usadel B (2014). Trimmomatic: a flexible trimmer for Illumina sequence data. Bioinformatics.

[75] Grabherr MG (2011). Full-length transcriptome assembly from RNA-Seq data without a reference genome. Nat. Biotechnol..

[76] Pertea G (2003). TIGR Gene Indices clustering tools (TGICL): a software system for fast clustering of large EST datasets. Bioinformatics.

[77] Li H, Durbin R (2009). Fast and accurate short read alignment with Burrows-Wheeler transform. Bioinformatics.

[78] Conesa A (2005). Blast2GO: a universal tool for annotation, visualization and analysis in functional genomics research. Bioinformatics.

[79] Finn RD, Clements J, Eddy SR (2011). HMMER web server: interactive sequence similarity searching. Nucleic Acids Res..

[80] Delorenzi M, Speed T, An HMM (2002). model for coiled-coil domains and a comparison with PSSM-based predictions. Bioinformatics.

[81] Stamatakis A (2014). RAxML version 8: a tool for phylogenetic analysis and post-analysis of large phylogenies. Bioinformatics.

[82] Magalhães DM (2016). LRR-RLK family from two *Citrus* species: genome-wide identification and evolutionary aspects. BMC Genomics.

[83] Fischer S (2011). Using OrthoMCL to assign proteins to OrthoMCL‐DB groups or to cluster proteomes into new ortholog groups. Curr. Protoc. Bioinformatics.

[84] Anders S, Pyl PT, Huber W (2015). HTSeq—a Python framework to work with high-throughput sequencing data. Bioinformatics.

[85] Love MI, Huber W, Anders S (2014). Moderated estimation of fold change and dispersion for RNA-seq data with DESeq2. Genome Biol..

[86] Vera Alvarez R, Pongor LS, Mariño-Ramírez L, Landsman D (2019). TPMCalculator: one-step software to quantify mRNA abundance of genomic features. Bioinformatics.

